# Exploring the experiences of leprosy stigma among patients and healthcare workers in Norte de Santander, Colombia

**DOI:** 10.1371/journal.pgph.0003939

**Published:** 2025-03-18

**Authors:** Carlos Mayoral-García, Anil Fastenau, Cristian Ghergu

**Affiliations:** 1 Faculty of Health, Medicine and Life Sciences, Maastricht University, Maastricht, The Netherlands; 2 German Leprosy and Tuberculosis Relief Association (GLRA/DAHW), América del Sur Region, Bogotá, Colombia; 3 German Leprosy and Tuberculosis Relief Association (GLRA/DAHW), HQ, Wuerzburg, Germany; 4 Marie Adelaide Leprosy Centre, Karachi, Pakistan; 5 Department of Global Health, Institute of Public Health and Nursing Research, University of Bremen, Bremen, Germany; PLOS: Public Library of Science, UNITED STATES OF AMERICA

## Abstract

This study examines leprosy-related stigma among patients and healthcare professionals in Colombia. Leprosy, classified as a WHO-listed NTD, is a chronic nerve disease causing sensory loss, disabilities, and deformities when untreated. This contributes to stigma, reducing quality of life, healthcare access, and income. Despite Colombia achieving WHO’s prevalence goal, some regions still face high detection rates. As a result, leprosy remains a challenge due to an incomplete understanding of the complete disease burden and its intertwined factors. The study consisted of 25 interviews with patients and healthcare workers, and field visits in Colombia’s Norte de Santander Department. Employing a constructivist approach to contextualize leprosy in Colombia through historical and socio-economic factors we integrate participants’ perspectives to enable flexibility beyond psychology’s rigid stigma categories and the disease’s narrow focus. Our research findings confirm regional research on patient stigmatization, including anticipated, internal, and experienced stigma, with a particular focus on the structural level and intersectional factors. This stigma becomes apparent when examining the organization of the healthcare system, the allocation of resources for leprosy prevention, diagnosis, and treatment, and the inadequate attention to patients’ mental health. Furthermore, we describe the commercialization of healthcare in Colombia, which perpetuates this situation by undermining the previously established leprosy community network, reducing the disease to a mere bacteriological perspective, and silencing patient narratives. Our research provides valuable insights for enhancing leprosy case detection, diagnosis, treatment, and social inclusion, ultimately improving patients’ quality of life. Recommendations for Colombia’s public health policies include involving patient expertise in leprosy programs, enhancing national clinical history systems, implementing active case detection, tailoring treatments to local contexts, and encouraging patient participation in comprehensive public initiatives. These measures empower patients, positively impact their mental well-being, and combat the stigma entrenched in Colombian society and institutions.

## 1. Introduction

Leprosy is a chronic infectious disease classified by the World Health Organization (WHO) as one of twenty Neglected Tropical Diseases (NTDs) and it is caused by a bacteria called *Mycobacterium leprae* that affects peripheral nerves, skin, eyes, and mucous membranes [1]. The presence of the bacilli within skin cells causes dermatological manifestations and, when untreated, the infection of the nerves leads to sensory loss, disability, and deformity [2]. In many cases, the diagnosis comes late due to stigma, lack of awareness, or healthcare-related issues contributing to physical impairments, posing a significant challenge for patient rehabilitation [3].

Two decades ago, Colombia achieved the WHO’s prevalence goal of 1 case per 10,000 inhabitants, the criteria for no longer considering leprosy a public health issue. However, certain Colombian regions have remained endemic, with Arauca and Norte de Santander exhibiting detection rates of 4.73/10,000 and 3.86/10,000 respectively [4]. The modest count of national cases, numbering 272 in 2022 [1], contributes to inadequate public funding, limited research, a dearth of experts, and insufficient healthcare facilities. Consequently, a prevalent issue of delayed diagnosis persists, leading to 30% of new cases manifesting disabilities and 10% experiencing Grade 2 impairments. In the context of endemic areas within Colombia, two studies have been conducted. The first, by Gomez et al. in 2020 [5], evaluated the stigma associated with leprosy, uncovering that 49% of participants experienced mental distress, and 27% faced participation restrictions. Further exploring the dimensions of prevalent stigma, another study conducted in the same regions revealed that close to 70% of leprosy patients are dealing with substantial anticipated and experienced stigma. This detrimentally affects their psychological well-being and hinders their social integration, thereby exacerbating the overall burden posed by the disease. Leprosy stigma is often observed within family, friend, and neighbour circles [6]. However, studies conducted in Colombia and other countries [6–8], indicate that healthcare providers also contribute to patient stigmatization.

With this research, we want to gain qualitative insights into leprosy stigma with a critical focus on the socio-cultural, historical, economic, and political contexts in which stigma is produced. Through this qualitative exploration, we aspire to unveil alternative pathways that diverge from conventional academic norms, as demonstrated in the work of van Brakel et al. [9]. Their approach advocates for comprehending health-related stigma as a universal construct, applicable across diverse cultures and health conditions. This stance facilitates the application of a standardized stigma concept, enabling data comparison and fostering global collaboration. However, while this approach streamlines data analysis and international cooperation, it also oversimplifies the complex issue and their evaluation is often confined within narrow parameters, employing standardized tools and scales that fail to encompass the entirety of the disease’s impact.

Similar methods have been employed to elucidate stigma in our research area [10]. However, this approach falls short of providing comprehensive insights into stigma, as it mainly confines itself to description and measurement rather than offering a holistic understanding. Another study undertaken in Colombia [6] employed focus groups and semi-structured interviews, although their analysis and findings remained confined within the overarching framework of stigma conceptualized by van Brakel et al. [9]. In light of this situation, a noticeable research gap emerges within the existing literature, highlighting the need to enhance our understanding of stigma in this specific region. Many studies on leprosy stigma underrepresents intersecting social identities like caste, class, and gender, often simplifying complex social experiences, reinforcing the need for a more nuanced, ethnographic approach to fully address the issue [11].

Only by obtaining a comprehensive perspective of the intricate interplay and repercussions of leprosy in patients’ lives, we can effectively elaborate interventions towards stigma eradication and patients’ complete recovery. Therefore, it is imperative to challenge the dominant conceptualization of stigma found in literature. This entails considering local factors and evading an isolated understanding of stigma.

The primary objective of our study was to investigate leprosy-related stigma among participants. If identified, we aimed to understand how this stigma is experienced by both patients and healthcare practitioners. By exploring health-related stigma, we intended to gather insights to inform future interventions in the country. Therefore, the research question that guided this study is as follows:

### How is leprosy stigma experienced in endemic areas of Colombia?

Sub-questions include:

- *How do patients experience leprosy stigma?*
- *How do healthcare workers experience leprosy stigma?*
- *What social, political, and economic contexts contribute to stigma?*

## 2. Methodology

### 2.1. Study design

The definition of stigma used in this study is, as stated by Weiss et al., “a social process or related personal experience characterized by exclusion, rejection, blame, or devaluation that results from experience or reasonable anticipation of an adverse social judgment about a person or group identified with a particular problem” [12].

The fundamental theoretical foundation of this research draws from a constructivist perspective on science and technology, as articulated by Bijker [13], and finds its inspiration in Diana Obregón’s [14] seminal work on the social construction of leprosy in Colombia. By embracing this perspective, we aspire to dissect the intricate dimensions underpinning the construction of stigma, unveiling the complex network of dynamics that influence its evolution within the context of leprosy.

Through the constructivist perspective, we used two models widely embraced in academia due to their applicability and user-friendly nature: the Socio-Ecological Model [15] and the Health-Related Stigma Model [9]. In applying the Health-Related Stigma Model, we developed themes surrounding anticipated stigma, self-stigma, and experienced stigma, which provided a clear framework to explore individual-level and interpersonal dynamics of stigma in leprosy-affected individuals.

**Fig 1:**
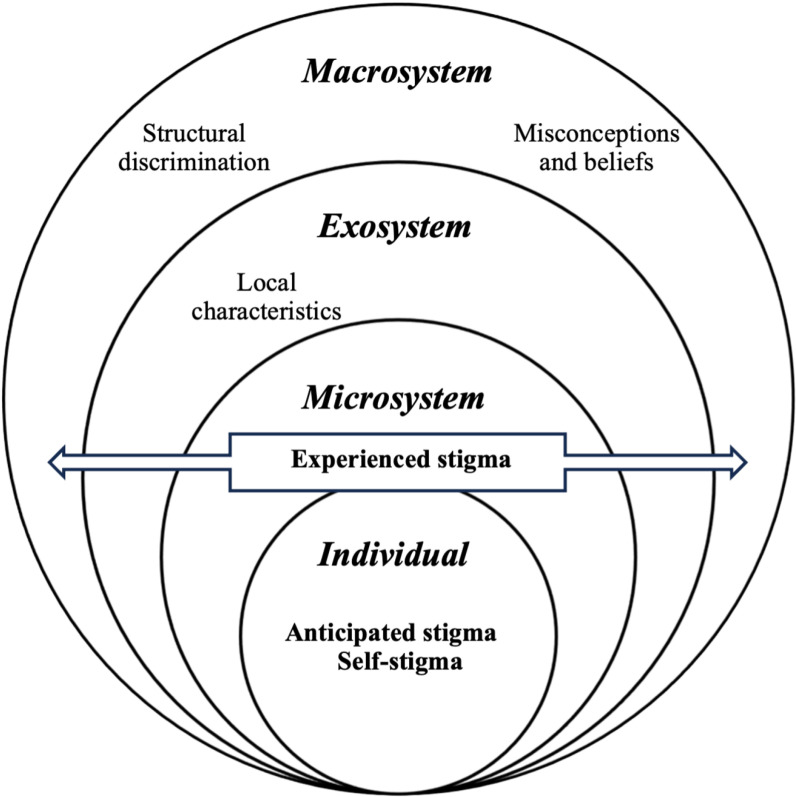
Socio-Ecological Health-Related Stigma Model.

To amplify the scope of this analysis, we incorporated the Socio-Ecological Model, allowing us to contextualize stigma within broader societal structures. This model added a macrosystem layer that captured the larger influences on stigma, specifically addressing structural discrimination, misconceptions and beliefs, and local characteristics of the region. These additional themes allowed us to examine how societal and environmental factors shape the perception and experience of stigma in this particular context. In Fig 1 below, we can see how interconnected both models to serve our analytical purposes.

Our constructivist approach involves a dynamic interpretation of this knowledge and encourages us to engage with these models actively rather than passively adopting them. Rooted in these principles, we contextualize these models within practical realities, recognizing them as constructs that offer utility in guiding individuals through their environments.

### 2.2. Study setting and participant selection

The study was conducted in Northern Colombia’s Department of Norte de Santander, that region was selected for being considered an endemic area with a detection rate of 3.86/10,000 [4]. Among the twenty-five conducted interviews, twenty participants had completed their treatment, enabling them to provide a comprehensive account of their experience with the disease. Moreover, two of them were actively engaged as social workers in leprosy campaigns. Recognizing the previous research [7,8] which identified stigmatization by healthcare providers, we included a small sample of healthcare workers to gain insights into daily patient care practices: five interviews were conducted with experienced healthcare professionals, including a chief nurse in a health center, two dermatologists, a social worker, and a leprosy coordinator. The majority of participants were aged above 40, with 17 women and 8 men. The interviews with patients were conducted within their residences, while those involving healthcare workers took place in private rooms.

### 2.3. Data collection

The participants were selected through purposive sampling, encompassing exclusively adult individuals (18 years and older) residing in the area under study, patients with confirmed diagnosis of leprosy and healthcare workers. The sampling strategy aimed for heterogeneity, encompassing a diverse range of individuals from various backgrounds to ensure a comprehensive understanding of lived experiences. To achieve this diversity, participants were enlisted with the assistance of social workers from the Norte de Santander Institute of Health, considering their willingness and availability. The recruitment period was from May 18, 2023, to July 3, 2023, and all participants gave written informed consent.

The interviews were personally conducted by the primary researcher in Spanish, his native language. However, to establish trust and cultivate a strong connection between the participants and the researcher, an experienced local social worker and well-regarded within the community facilitated the introductions.

The interviews had a duration of approximately 30 to 90 minutes and were recorded in audio format. Following the introduction and obtaining informed consent, the interview commenced under a pre-constructed guide (S1 Table) containing open-ended questions, encouraging participants to provide detailed responses. In response to emerging insights, revisions were applied to the topic guides accordingly. The number of interviews was determined based on the research objectives and the availability of participants within the leprosy patient population. Data collection continued until it was clear that no new themes or insights were emerging, signalling that data saturation had been reached. We ensured trustworthiness by exploring the perspectives and experiences of multiple actors: patients, healthcare workers, and social workers. Additionally, confirmability was maintained through reflexivity, with the researcher continuously reflecting on potential biases and seeking feedback from peers and local collaborators to ensure objectivity.

### 2.4. Data analysis

After transcribing interviews and becoming familiar with the data, we employed thematic content analysis [16] to organize and synthesize emerging topics. The theoretical framework we adopted served as a starting point, however, we approached the data with a constructivist perspective, allowing themes, categories, and codes to emerge in a deductive manner, leading to richer data and a broader understanding. While deductive approaches often rely on predefined themes, we recognized the need for inductive coding to capture the nuances of participants’ experiences, facilitating a more comprehensive analysis of the data.

For each theme, we developed detailed categories and codes [17]. Codes were refined for specificity and to prevent repetition. Categories were cross-referenced with color-coded themes, aligning both with our theoretical framework and the topics emerging from the interviews. The full list of categories under each theme, along with the main codes, can be found as a table in the supporting information (S2 Table). Throughout data collection and analysis, the researcher remained mindful of their preconceptions, social context, and broader influences on the data.

### 2.5. Ethical considerations

Given the sensitive nature of the disease under investigation, we placed special emphasis on ensuring voluntary participation and addressing concerns related to coercion [18]. To ensure comfort and maintain confidentiality, participants were given the freedom to select a private setting for the interviews. Additionally, the study’s objectives and privacy measures were comprehensively explained, providing participants with the opportunity to seek clarification and ask questions. All participants provided informed written consent. While conducting research in Colombia, we acknowledge the historical colonial context and the implications of a Spanish researcher’s presence. Therefore, our study incorporates specific considerations, including cultural sensitivity, collaboration with local experts, respect for participants’ autonomy, reciprocity, transparency, ongoing reflection on researcher positionality, and open dialogue to address ethical concerns. These practices ensure an ethical and respectful research process. Ethical approval was obtained from the ethics committees of both Maastricht University in the Netherlands and the University of Santander in Cúcuta, Colombia, registration number CEI-ISEM-02-2023.

## 3. Results

This qualitative study includes 25 semi-structured interviews conducted from May to July 2023 in the region of Norte de Santander, Colombia. Employing the stigma conceptual model proposed by van Brakel et al. [9], we were able to build on the findings presented by van Wijk et al. [6], particularly in relation to the presence of various forms of stigma within the Colombian context. The prevalence of anticipated stigma was evident among our participants who refrained from divulging their condition to family and acquaintances, often resorting to excuses or concealing their illness through various means. Internalized stigma, on the other hand, was less frequently encountered, as many individuals possessed a comprehensive understanding of leprosy. Nonetheless, a prevalent feeling among participants was a weakening of self-assurance and a feeling of diminished worth due to the social and occupational constraints brought about by the disease. Therefore, the experience of stigma was predominantly triggered when patients chose to share their diagnosis with others. While family members tended to provide support, instances of discrimination typically arose from the community and healthcare workers. However, as we will explore later, it is important to recognize that a substantial source of experienced discrimination stemmed from a structural level, interlinked with the way the healthcare system is organized and the prevailing perception of the disease.

The interviews shed light on the stigmatization and neglect that the leprosy burden entails, underscoring issues within the health system’s organization, resource allocation for prevention, diagnosis, treatment, the attention given to patients’ mental well-being, and the challenges that healthcare workers face in practice. Furthermore, our analysis explores prevalent trends, notably the commercialization of healthcare. This trend perpetuates the situation by undermining the historical leprosy community network, narrowing the disease’s understanding to a mere bacteriological viewpoint, and silencing the narratives of patients.

The results section is divided into three chapters, each addressing key themes identified in the study. The first chapter, Patient’s Journey, follows the progression of leprosy as described by the patients, from symptom onset through diagnosis, treatment, and recovery. This structure mirrors how the interviews naturally unfolded, making the narrative more engaging and easier to follow.

The second chapter focuses on mental health, a recurring theme in the interviews. Given the lack of mental health resources in the region and the significant emotional burden faced by leprosy patients, we felt it was essential to dedicate a separate chapter to this issue.

The final chapter examines structural discrimination, the most critical theme in our research. It explores the systemic barriers and societal stigmas that affect leprosy patients, emphasizing its profound impact on their experiences.

### 3.1. Patients’ journey

In the results section, we navigate the journey of both healthcare workers and patients through the challenges posed by the Colombian health system. The structure of this section has been organized around key stages of the disease: diagnosis, treatment, and recovery. It is important to note that while we present this journey in a structured manner, the reality is far from linear due to its complexity. For instance, patients may encounter misdiagnoses leading to treatment changes or relapses, illustrating the intricate nature of their experience. Moreover, this journey is not confined to a specific timeframe or location, as our data includes insights from individuals who have undergone various leprosy treatments or management programs.

Our study compares leprosy management from past to present amidst significant societal and political shifts. Participants who experienced these changes highlight the evolution from a cohesive healthcare community to a fragmented structure, as seen in their references to the support provided in the past by the IDS throughout their leprosy journey,

Before, you could go to the IDS, have a bacilloscopy done the same day, and after a week, return to pick up your medication. Everything was handled in one place, with no waiting times. (Patient 2)

the deterioration of the patients’ association

It [the association] has changed a lot because it is no longer useful (…) You go there, you sit down, they charge you and that’s it. That’s not an association. (Patient 2)

or the current health system divided among different providers.

The programs were vertical because the health authority managed everything: surveillance, testing, treatment, follow-up, and active case detection. They didn’t delegate these responsibilities to different healthcare providers as they do now. (HW 2)

The introduction of multidrug treatment shortened therapy but caused side effects like skin darkening. Another pivotal change was the shift from vertical programs, overseen by the Health Departmental Institute (IDS), which managed surveillance, testing, diagnosis, treatment, and active case search, to a decentralized system. Under this new structure, private institutions, known as Health Promoting Entities (EPS), administer healthcare services to the population through a network of public and private clinics, known as Health Providing Institutions (IPS). This complex system operates on contributions from employed individuals and offers subsidies for the unemployed. Presently, the IDS plays an advisory role, ensuring protocol adherence. Other stakeholders include the weakened yet active patients’ association and the former leprosariums of Agua de Dios and Contratación, which now provide subsidies and nursing homes. Nonetheless, contemporary challenges include extended waiting times, staff turnover, administrator bankruptcies, corruption, lack of widespread knowledge about available services, healthcare commercialization, loss of clinic history, internal migration, and EPS affiliation. The description given by an experienced dermatologist illustrates this situation:

It is a kind of commercialization (…), they [the EPS] also need to make money. (...) What happens is that the EPSs are playing games so that people do not get sick. (...) The doctor sends him an MRI, then either they say “Wait, better have it looked at by someone else” or they delay it. There are three months of wait. Then, when they are going to give the MRI, the patient is even more screwed, or if he has died, then they already saved from paying for the MRI. (HW 3)

#### 3.1.1. Diagnosis.

During our interviews, we observed three main scenarios in the trajectory of individuals with leprosy, which often begins when they notice unusual skin discolorations. They either resort to self-diagnosis and use over-the-counter creams, ignore the issue altogether, or seek an appointment with a general practitioner.

Some patients visit the doctor, while others remain indifferent and choose not to seek any treatment. (…) Others are discouraged by the long waiting times and opt for self-diagnosis, going to a pharmacy and purchasing a cream. (HW1)

When patients do consult a doctor, the diagnosis process becomes intricate due to challenges such as inadequate attention to leprosy, communication issues, and other complicating factors.

There are other diseases that also affect the skin and the nerves that are not leprosy. So, you must, inside the scientific knowledge, be able to differentiate from other pathologies. (HW 3)

As a result, a significant portion of our participants received incorrect diagnoses and subsequently underwent treatments for different diseases such as mycosis, eczema, dermatitis, psoriasis, or allergies. Most of them get a correct diagnosis when they start experiencing a loss of sensation in their extremities and they are scheduled for a bacilloscopy and a biopsy. However, due to lengthy waiting lists, this process can span several months, reflecting the considerable average delay in Colombia, which stands at 2.7 years [10]. Interestingly, neither patients nor doctors anticipate a leprosy diagnosis, as it is widely perceived as a disease of the past, commonly believed to be eradicated:

When I tell my family that I have a [leprosy] patient they all say: What? Really? I thought that didn’t exist anymore. (HW1)

The process described is complex and prolonged, exacerbated by initial oversight of leprosy. Multiple doctor appointments compound the challenges, requiring patients to repeatedly visit health centers, causing personal and financial strain. During this time, nerve damage worsens, leading to frustration and distrust in the healthcare system. Some patients blame doctors for overlooking leprosy, despite family history suggesting it. Such frustrations can impede treatment adherence and acceptance of home visits due to diminished trust. This distrust affects doctor-patient interactions, with patients often avoiding discussing leprosy with doctors for fear of biased diagnoses based on their past history with the disease.:

I don’t like going to the doctor because, I mean, they are always going to relate everything to that [leprosy]. So, I never mention it. (Patient 13)

It has been previously highlighted that Stigma is a socially constructed concept that takes on diverse connotations based on context. In this particular environment, stigma is shaped by the local context, thus differing from those associated with other illnesses and situations. Here, the patient’s interpretations of the disease significantly influence the perception of stigma, as their explanations serve as a rationalization. In cases where scientific understanding is incomplete or not comprehensible, patients construct their sense of understanding. Leprosy is often regarded as an enigmatic disease, fostering room for alternative explanations that might be categorized as misconceptions within medical discourse:

I think that this disease is very mysterious (…) because I see and meet people who never got it even though they lived with affected people, and then people who never met a patient and appeared with the disease, this is a mystery. (Patient 2)

Frequently, participants shared experiences of having relatives with leprosy. However, in instances where patients lacked such connections, establishing causality was problematic:

I say that I caught my disease because I helped my husband, we used to burn coal, so I say it was that. I had to take out the coal from the fire with a stick and then load it in a car to sell it and that stuff, with products of that kind (…). Yes, I say that it had to be the heat and that. (Patient 10)

Patients frequently find significance in the disease by seeking explanations rooted in their own circumstances, associating it with prior employment in hazardous conditions. The concealed nature of leprosy contributes to an aura of uncertainty around its origins, leading those unfamiliar with the illness to adopt alternative interpretations. The attribution of leprosy to living in unfavorable environments and other societal factors alters the stigma from being contagious to being indicative of poverty. Moreover, certain participants endeavored to lessen the impact of the disease by erasing the boundaries between patients and healthy individuals:

I don’t know if is true or a lie, but people say that practically we all have the disease bacillus (…) but it simply scales up in one and not in others, so why are we going to discriminate against someone for any sickness? (Patient 2)

Participants often attribute their infection to factors such as border migrations with Venezuela, immunological vulnerabilities, and dietary practices. Interestingly, broader structural influences impacting disease control efforts in the country, such as limited health coverage and weak law enforcement in remote areas, or a lack of coordination in border regions, often remain unspoken. These intertwined factors are closely interlinked with stigma and are subject to contextual shaping. Notably, in line with other studies [19] that indicate a strong association between direct contact with wild armadillos and an increased risk of leprosy, some participants—including patients, healthcare workers, and dermatologists—identified the armadillo as a potential transmission vector. While some expressed doubts about its veracity, they still deemed it important for public health:

The person that eats much armadillo contracts the disease because the armadillo has that disease. The people from Ocaña eat it a lot. And there are many patients there. So, you should tell people to not eat that. (Patient 2)

Another factor is religion, which serves as a foundational cornerstone not only in the historical context of leprosy but also within Colombian society, playing a significant role in how the disease is interpreted. Contrary to being a source of conflict or discrimination, participants frequently invoke religion to make sense of the disease’s origin and progression. Even when questioned about their beliefs regarding why God permits leprosy, religion emerges as a framework through which they seek understanding:

God gave the skills to Doctor Hansen, who discovered the bacilli and made the drugs. (…) He consents the disease in order to improve science or to have more research, so they find the cure. (Patient 2)

Healthcare workers hold their own perspectives on leprosy, influenced by its minimal inclusion in their training and infrequent encounters throughout their careers, resulting in its eventual neglect in practice. The lack of emphasis on leprosy within their professional education contributes to the spread of stigma in this context, subsequently influencing their future interactions with the disease. This was particularly evident in the patients’ explanations, where encounters with doctors who exhibited an aversion to touching or struggled to explain the disease resulted in discriminatory experiences.

Some long-term leprosy patients possess a deep understanding of the disease, often surpassing that of some doctors. Their knowledge spans from medical procedures like bacilloscopy to treatment regimens and historical developments. Actively engaged in medical discussions, they offer invaluable insights derived from decades of firsthand experience, contributing to the detection of new cases and providing empathetic support to fellow patients. Their empathy, stemming from shared experiences, helps alleviate patient apprehensions, especially regarding public stigma. Embracing their expertise not only fosters inclusion but also empowers them with a meaningful role in patient care.

When she [the patient] hugged me, she said: I love that you did it [the visit]. That’s what I like, I like it when I do the visits because sometimes there are many questions, and I can answer the patient all of them. (Patient 2)

This approach proved highly effective in the past, as dedicated leprosy promoters accompanied patients throughout their entire journey with the disease. This success was attributed to greater resources allocated to leprosy programs, a more paternalistic approach, and prompt and comprehensive action encompassing all essential aspects of diagnosis, treatment, and recovery.

The advantage was that they saw the patient but also at the same time the family members, and the social conditions, provided treatment and made sure the patient took the drugs. They considered at the same time the level of disability and applied for the disability subsidy. (HW 4)

Local structural factors pose significant barriers to sustaining this practice due to changes in healthcare organization. Many patients, predominantly low-income earners, rely on subsidized healthcare, which prohibits assuming roles as leprosy social workers or any other job unless they stop being subsidized and begin paying for health insurance. The absence of official transportation from the IDS adds to the disincentive, requiring personal expenditure for work-related costs. Consequently, patients feel marginalized in program design, highlighting contextual factors in shaping stigma. Their reluctance to engage stems from structural obstacles rather than anticipated stigma. During field visits, individuals displaying leprosy symptoms approached us for information, underscoring the need to enhance prevention strategies and questioning prevailing incidence rates.

The diagnosis phase stands out as one of the most challenging aspects of dealing with leprosy. The lack of awareness about leprosy among both medical practitioners and patients, along with false negative results, erroneous diagnoses, physician burnout leading to quick and inadequate care, and patients’ reluctance towards doctors and biomedical approaches, collectively contribute to delayed diagnoses. Typically, by the time the diagnosis is established, patients are already experiencing reduced sensitivity and irreversible nerve damage.

#### 3.1.2. Treatment.

In Colombia, leprosy treatment is provided free of charge, with patients required to visit healthcare facilities monthly for medication. However, accessing treatment poses challenges. Many patients, seeking to avoid stigma, prefer home delivery or undisclosed treatment locations. Yet, home delivery is impractical due to limited IDS resources and travelling longer distances imposes financial strains on patients, often causing interruptions in their treatment.

Furthermore, in remote regions like northern Norte de Santander, particularly near Tibú, accessing healthcare is even more challenging due to the area’s history of conflict and illicit activities. Designated as a Red Zone (areas with frequent conflicts between official forces and insurgents), entry and exit require permits, adding to the complexity. Residents face daily hardships, including displacement and loss of family due to ongoing conflict. Stigma for patients in this context is deeply intertwined with these challenges, impacting every aspect of their lives and treatment adherence. The situation is exemplified by an incident where a paramilitary group used force to ensure a patient continued treatment:

A while ago a positive patient took the first blister of the treatment and then said that he wasn’t going to continue. Do you know what the “paracos” [paramilitary] did? Executed. They shoot him. (HW 5)

The public insecurity situation encompasses further interrelated factors, including the challenges faced by patients working as coca farmers. The government’s initiatives and the conflicting interests of various groups have resulted in a decrease in coca demand, leaving these farmers without a stable income. This, in turn, adversely impacts their capacity to access the necessary treatment:

So now, as the coca is not giving them enough, not even for food, the patient is not coming for the treatment, and of course not visiting the doctor. (Patient 2)

Hence, the degree of stigma’s impact on a patient varies depending on their specific context and environment. Factors such as public insecurity, unstable income, or geographical remoteness can lead to distinct experiences of stigma, setting these individuals apart from more fortunate patients.

Participants frequently mentioned experiencing secondary effects from the treatment, including fatigue, disrupted sleep, weight loss, reddened urine, leg inflammation, and skin darkening. These physical changes often lead patients to withdraw from social interactions, influencing stigma. However, the full impact of these treatment-related effects on stigma is often overlooked. Besides the visible changes that provoke shame, patients also struggle with reduced physical strength, limiting their engagement with the outside world. This dual impact reinforces their isolation, intensifying the stigma they face.

#### 3.1.3. Recovery.

A patient is considered free of the disease when the bacilloscopy returns a negative result, and subsequent monitoring occurs annually for a span of a few years. However, leprosy encompasses dimensions beyond the mere bacterial count, exerting ongoing effects on various facets of life that persist after treatment. This oversimplified perception of the disease proves considerably problematic from the patients’ standpoint, giving rise to perplexity and exasperation. The narrative of one participant notably underscores how the term “cured” carries divergent implications for healthcare workers and patients:

If I am cured, why if I stop, I sprout again? (...) because I can’t say that I am sick or that I am cured, because only them [doctors] are the ones that know, right? However, it is me who is feeling the disease, right? (Patient 18)

Furthermore, post-treatment, patients often require ongoing wound care, specialized footwear for foot deformities, and must secure a livelihood. However, many face challenges in returning to their previous occupations due to resulting disabilities. The requirement for clinical assessment before employment, necessary for insurance coverage, forces individuals to disclose personal information to strangers, a discomforting prospect for those who avoid discussing leprosy even within their families. Additionally, physically demanding job roles often disqualify them from passing the examination. Consequently, many participants turn to self-employment as their only option to sustain their households.

As emphasized by a healthcare worker, various factors can influence the need for a disability subsidy for a patient, including situations involving domestic violence. Such circumstances can significantly impact the assessment and decision-making process concerning subsidy approval, as exemplified by the experience of this particular patient:

Her husband was treating her badly. She used to say that she had to keep living with him because she couldn’t work [because of a disability], so we gave her a subsidy. She is always so grateful, and she broke up with that man. [Domestic violence] was complicating her situation and she was also in constant depression because of her situation and the disease. (HW 4)

This case illustrates why the rigid classification of the disease and the criteria for granting a disability subsidy may not always adequately address individual circumstances. It underscores the importance of adopting a more adaptable and inclusive approach. The impact of intersecting factors in a patient’s life can significantly alter how stigma is experienced, necessitating a nuanced consideration. However, achieving this entails providing personalized care and maintaining close engagement with the patient.

Finally, a crucial part of leprosy recovery is patients’ social integration. Patients’ associations play a key role by educating members, disseminating information, fostering connections, and organizing social activities. They also provide skill-building opportunities, like craft-making, and support small business ventures, though challenges, like participants selling businesses for personal gain, have arisen.

However, in recent years, the patients’ association has shifted from its supportive role to become a financial burden for some members. Monthly contributions, coupled with increasing transportation expenses, strain patients already dealing with disabilities and requiring assistance. Additionally, the association has begun charging fees for survival certificates, originally meant to aid disability subsidy applications, but now contributing to discrimination and unintended negative outcomes.

People go to the association out of obligation, fearing that their disability subsidies will be revoked if they don’t (…) They are charged 5,000 pesos for a certificate they shouldn’t be charged for. Additionally, some individuals need to be accompanied by family members due to being in wheelchairs, which adds an economic burden on the family. (Patient 2)

### 3.2. Mental health

The healthcare system currently lacks mental health support and struggles to allocate time for monitoring patient well-being. In contrast, IDS staff go beyond their roles, offering assistance with subsidies, diagnoses and mental health assessments. Despite criticism for their paternalistic approach, this close relationship fosters positive mental health outcomes, reflecting the challenges of the current system’s inability to provide personalized care.

It is hard for you to detach yourself from the patients and say to them “no look, we can’t see each other here, because the regulations say we can’t”. (HW 4)

Parents among the participants expressed deep concerns about transmitting the disease to their children and experienced a loss of interest in engaging with them, preferring isolation. Alarmingly, one participant even attempted suicide, emphasizing the complexity of addressing mental health needs. Financial barriers to accessing medication and psychological support further exacerbate these challenges.

Once I tried to take my own life and I took a bottle of sleep drops. I tried everything. What I wanted was to fall asleep, not wake up again. (Patient 17)

The dearth of job prospects and the quest for social acceptance significantly impact patients’ mental well-being. Following diagnosis, individuals often lose their usual outlets for diversion, like work and social engagements, leaving them isolated at home, grappling with feelings of insignificance. Lack of open discussions about the disease within families further compounds this isolation, resulting in a muted social life for patients. We encountered families with multiple members affected by leprosy, where coping mechanisms resembled closely guarded family secrets. In some cases, the disease’s prevalence within a family even tarnished its reputation in the community. Growing up in such environments can shape individuals into reserved, introverted personalities content with homebound lifestyles.

I was always shy (…) My mother always kept me at home (…) Our family [affected by leprosy] has always been very reserved, and we don’t have many friends (…) We prefer to invite family over to our home to celebrate. (Patient 3)

### 3.3. Structural discrimination

Our interviews revealed numerous instances of discrimination and stigmatization, from partners, family members, friends, neighbors, employers, or healthcare workers. However, what we found particularly interesting is how discrimination can be traced to broader systemic levels, because this adds a new layer to previous research and underscores the necessity of avoiding a narrow focus when developing interventions to combat stigma.

Through the patients’ journey, it becomes evident that discrimination is deeply embedded within the structure of the healthcare system, exerting its influence on the diagnosis, treatment, and recovery of the patient. This prevailing discriminatory attitude is manifested in the lack of attention and significance accorded by the government in comparison to other diseases:

In the ministry, when they do coordinators meetings of each department [leprosy and tuberculosis meetings], there is discrimination because the talks are short, and all the time is given to tuberculosis. (HW 4)

The structural changes that have occurred in the healthcare system over the past few decades serve as a clear example of how leprosy is not considered a priority or a responsibility, as the EPS has taken over the role. The same organization of care also reveals its drawbacks in long-term interventions in rural areas, as these regions are often neglected and overshadowed in comparison to urban populations.

Because of the personal rotation and people doing the rural year [mandatory year of practice in rural areas for HW], they can spend one year in a village. But it finishes and when they are learning they remove them. (HW 4)

Throughout our interviews with healthcare workers, a prevailing feeling of burnout in their roles emerged, which they identified as a significant issue impacting patient diagnosis. They also expressed dissatisfaction with the leprosy training, as it was conducted during their working hours and transitioned to online platforms during the COVID-19 pandemic. These healthcare workers highlighted the disparity between the ideal scenario and the practical challenges they faced. Specifically, when it comes to diagnosis, they described difficulties in communicating with the IDS and feeling unsupported in those critical moments.

The system is that the doctor is given four patients in one hour. So, the consultation of each patient is 15 minutes and sometimes it is not enough, because if he doesn’t get confident with you, he is not going to tell you anything. (HW 4)

The healthcare worker’s testimony highlights the hurdles in accurate diagnoses when patient backgrounds are unfamiliar. This underscores the limitations of training or awareness campaigns alone in rectifying misdiagnoses. The intricate interplay of local context and healthcare system organization shapes Colombia’s stigma landscape, warranting a holistic approach to address misdiagnosis issues. Chronic underfunding not only destabilizes programs but also leads to an unstable work environment, seen in reliance on temporary contracts even for IDS staff. Moreover, the low leprosy incidence complicates matters, making it hard to garner attention to these concerns and potentially leading to under-detection without active patient identification efforts and field experts to validate statistics.

Leprosy’s intersection with politics is notable, with debates extending to subsidy amounts, even becoming election promises. Patients historically engaged in political activities through their association, but its recent decline leaves them unrepresented and their concerns unheard. This loss of representation silences the patient’s voice, highlighting structural discrimination faced by those affected by leprosy.

## 4. Discussion

Our findings aligned with earlier studies conducted in the same region [6] regarding the presence of stigma among patients and healthcare workers. Nonetheless, our research has unveiled an additional facet for comprehending leprosy-related stigma. Throughout this study, we have introduced a renewed perspective that underscores how structural factors can intricately shape how stigma is encountered. This approach has allowed us to delve deeper into the multifaceted nature of leprosy-related stigma, considering not only individual beliefs but also systemic elements that contribute to the formation of these experiences.

While previous research [6] has predominantly examined stigma across individual, relational, and communal domains, encompassing both anticipated and internalized stigma, our study examines deeper into experienced stigma. This kind of stigma, often observed within interpersonal relationships and communities, warrants an intensified focus on the intricate social mechanisms that underpin the perpetuation of leprosy-related discrimination within Colombia’s structural framework.

We chose to divide the discussion into three categories: commercialization, bacteriological reductionism, and patient knowledge. These categories emerged organically from the data, revealing how stigma is perpetuated within Colombia’s healthcare system.

Commercialization highlights the shift from social medicine to a market-driven healthcare model, which deprioritized leprosy due to its low incidence and lack of profitability. This neglect emerged as participants reflected on how leprosy programs have diminished over time, illustrating the structural changes affecting patient care.

Similarly, bacteriological reductionism reflects the disconnection between the biomedical model, where “cure” is defined by the elimination of bacteria, and patients’ lived realities, where they continue to suffer from long-term effects and social stigma. Our analysis expands on this disconnection and the barriers and struggles shared during the interviews that are often overlooked but are critical in understanding how leprosy stigma is experienced.

Lastly, patient knowledge bridges local beliefs with biomedical explanations. Participants frequently combined scientific and cultural understandings of leprosy which is crucial for understanding how patients make sense of their condition amidst stigma. This category also serves to encapsulate the alternative reasonings and explanations for leprosy shared by participants, shedding light on aspects of the disease experience that may otherwise be undervalued but are significant.

These insights underscore the ways structural factors shape the leprosy experience beyond individual and interpersonal stigma, warranting broader inclusion in the analysis of health-related stigma.

### Commercialization

From a broader perspective, it became apparent that the perception of the disease has undergone a process of commercialization, intertwined with the transformations in the healthcare landscape over the past decades. Up until the late 1980s, Colombia adhered to the Latin American trend of social medicine, resulting in one of the continent’s admirable healthcare ratings [20]. However, with the enactment of Law 100, which restructured the healthcare system into a regulated market of health insurance providers, the trajectory shifted towards the privatization of healthcare and a subsequent decline in accessibility to medical services [21]. This contemporary healthcare trajectory also reflects the country’s colonial past, influenced by the imposition of European health standards and professionalization as superior benchmarks [22]. This commercialization narrative could be indicative of a continuum of colonial power dynamics, where globalization processes and a preference for foreign health models have further perpetuated the trend.

Leprosy, characterized by its low incidence rate and the continuous need for human and financial resources for case identification and patient follow-up, has fallen victim to further neglect within the context of the healthcare system. This disregard can be attributed to the absence of commercial incentives for both the EPS and the IPS. Driven by a cost-benefit analysis, these entities tend to limit their services, shorten consultation times, and prioritize the needs of the healthy population over those of leprosy patients [23].

Our results align with other studies in Colombia [24] that underscore the significance of family leprosy transmission as a primary mode of disease spread. This finding reinforces the notion that closely monitoring household contacts serves as a valuable strategy for both early leprosy diagnosis and the surveillance of its transmission dynamics. However, the prevailing trajectory of the country’s healthcare system is not aligned with such proactive strategies. The current direction of the healthcare system does not prioritize these approaches, which could have a potential impact on leprosy control efforts. The findings emphasize the importance of bridging this gap between research insights and healthcare policies to effectively address the challenges posed by leprosy transmission and its associated complexities.

The transformation of healthcare into a commercialized sphere has also altered the approach to managing leprosy, shifting it from a charitable or paternalistic model to a more medicalized and impersonal form of care. Consequently, this transformation has led to a reduction in the scope of healthcare workers’ involvement, with less emphasis on continuous patient support throughout their journey with the disease. As a result, various facets of leprosy beyond the clinical aspects have been overlooked and forgotten, thereby undermining initiatives like the patient’s association mentioned in the results. It becomes apparent that this commercialized approach has contributed to the leprosy patient association losing its capacity to effectively represent patients and foster their inclusion, missing the connections that once bridged the gap between old and new patients.

Examining the participants’ past experiences with the association provides an interesting perspective. While the outcomes of stigma are commonly seen as negative, it is worth noting that positive outcomes can also emerge. Stigma has been acknowledged to foster resilience among marginalized groups [25], and it has played a role in catalyzing the establishment of patient advocacy groups and campaigns. These efforts have contributed to significant policy changes aimed at enhancing access to healthcare for stigmatized conditions, such as HIV [26]. In the case of leprosy, this was indeed the case in the past, challenging the oversimplified notion that vulnerable populations are solely defined and connected by their perceived vulnerability. This highlights the fluid and complex nature of how patients are conceptualized [27].

### Bacteriological reductionism

The contrast between the biomedical perspective of leprosy as a simple bacteriological count and the patients’ holistic understanding exemplifies the repercussions of the commercialization process. The eradication of the Hansen bacillus has led to the perception that a patient is no longer considered sick, overlooking the enduring effects of treatment, the persistent impacts of leprosy, and the ongoing challenges in accessing treatment and sustaining livelihoods. Consequently, the conventional binary notion of sickness and health becomes problematic when put into practice.

The patient’s refusal to fully embrace the concept of bacteriological cure is intrinsically tied to their resistance to being categorized as “socially cured”. This category was introduced by the Colombian state in the 1920s to label those individuals who were no longer considered a threat to society due to their improved health condition. Those falling under this category were discharged from leprosariums, and their subsidies were terminated, as they were expected to reintegrate into society. However, this reductionist approach to the disease was primarily driven by economic considerations to reduce the cost of maintaining leprosariums when confinement was mandatory [21,28]. The same conflicts and challenges from the historical leprosy context continue to reverberate in the present day.

The reductionist perspective rooted in bacteriological analysis is not limited to the recovery phase of the patient but also extends to the diagnosis process. In the pursuit of making biomedical techniques like biopsies and bacilloscopies work, other valuable methods like physical examinations and comprehensive medical history evaluation lose their significance. When these biomedical methods fail due to human error or prolonged waiting times (average diagnosis delay in Colombia stands at 2.7 years [18]), patients are left with no alternative for diagnosis, even if their symptoms are apparent to an experienced medical practitioner. Indeed, the failure to recognize and consider other dimensions of the disease beyond the mere bacteriological count, coupled with the disregard for the importance of intersectional factors in comprehending leprosy and its associated stigma, directly contributes to a system of structural discrimination. By neglecting to incorporate a broader understanding that encompasses the socio-economic, cultural, and contextual elements of leprosy, the health system inadvertently perpetuates disparities and barriers, ultimately deepening the impact of stigma and hampering effective interventions.

Overlooking factors like the patient’s geographical location or the local security situation can lead to a superficial understanding of how leprosy-related stigma is experienced. This approach can miss the intricate connections between leprosy stigma and broader forms of marginalization, such as those based on race, class, and gender. Aligning with previous research [26], our findings emphasize that leprosy stigma interacts with these other layers of disadvantage, resulting in varying degrees of vulnerability to health-related stigma. Our study echoes the findings [8] that highlight the impact of socioeconomic status on the experience of health-related stigma particularly emphasizing poverty as a significant intersectional challenge. Neglecting to account for these intricate factors may exacerbate the burden of leprosy, potentially leading to treatment disruptions and negative outcomes. To comprehensively address the complexities of leprosy’s impact, it is crucial to recognize and tackle the intertwined factors that contribute to stigma, guiding the development of interventions that address the multifaceted hurdles faced by patients.

Considering the historical struggles for professional authority among doctors in Colombia [22], it is interesting to observe how contemporary doctors prioritize maintaining their status of trust and reliability. This emphasis on credibility can be viewed as an extension of the ongoing legacy of medicalization, which, as Platarrueda Vanegas [28] highlights, remains incomplete due to broken dialogues and the exclusion of experiential knowledge. The medical narrative intersects with other dimensions of the disease in the patient’s understanding of leprosy. The concept of bacteriological reductionism [28], where a patient is considered cured solely based on treatment completion, contrasts sharply with the patient’s uncertainties and frustrations, as they don’t perceive themselves as fully healed. This is further exacerbated when the patient suffers from leprosy reactions. Unfortunately, now that multibacillary leprosy is largely treated with one year of multidrug therapy, reactional states may continue after the completion of therapy [29]. Therefore, the complex aspects of the disease create space for varied interpretations and alternative viewpoints that should be integrated alongside the biomedical approach.

Furthermore, the frequent misdiagnosis of leprosy by doctors who lack sufficient time to familiarize themselves with patients or who are frequently rotated to different clinics erodes confidence in their role. On the contrary, experienced non-professionals who possess a deeper understanding of leprosy often step in to bridge this gap in the system, due to insufficient training or burnout of doctors. These experienced patients become the primary sources of information and diagnosis for new patients. Consequently, the authority of doctors is undermined in such situations, leading to conflicts, and compromising effective communication with patients. This scenario underscores the importance of addressing the shortcomings in the medical system to enhance patient-doctor relationships and overall healthcare outcomes.

### Patient’s knowledge

Consistent with existing literature suggesting that patients adopt various explanations for leprosy causality to mitigate societal fear [30], our study reveals that participants also incorporate theories of heredity, lifestyle, nutrition, environment, and social conditions to contextualize the origins of leprosy. Interestingly, some participants attributed the disease’s onset to factors such as immunological deficiencies, unsanitary conditions, dietary habits, warm climates, or unhealthy occupations. This intertwining of biomedical and experiential narratives becomes evident when examining the origins of leprosy’s endemic nature. Healthcare workers also acknowledge immunological changes and unfavorable conditions as contributing factors to leprosy development, thus validating local beliefs. This convergence of local reasonings and biomedical insights demonstrates a complex interplay of explanations that seek to rationalize the occurrence of leprosy, offering a bridge between cultural interpretations and medical understandings.

Religion, historically intertwined with the perception of leprosy, continues to have a role in the contemporary understanding of the disease. Traditionally associated with sin and punishment, leprosy carried stigmatizing connotations [31]. However, our study did not reveal such negative associations in participants’ interviews. Instead, religion serves as a framework for making sense of leprosy, giving to God the authorship of scientific discoveries and seeing faith as a source of hope and comfort. This patient-driven interpretation of leprosy emphasizes the complexity of its cultural and medical perceptions, offering insights into the nuanced ways individuals contextualize and explain their experiences with the disease.

Incorporating the medical construction of stigma into our analysis led us to encounter certain situations that did not neatly fit established categorizations. For instance, participants’ hesitance to disclose their medical history to doctors could be perceived as a form of anticipated stigma by medical definitions [6]. However, our findings revealed that some participants chose not to disclose their leprosy history to doctors in order to secure a diagnosis unrelated to leprosy, taking advantage of the doctors’ lack of awareness. This behavior has been observed in obesity, where patients conceal information to avoid prejudices and the iatrogenic harm associated with an inaccurate diagnosis [32]. In this context, leprosy becomes entwined with other diseases, impacting the diagnosis process and the doctor-patient relationship.

From a patient’s standpoint, where time with the doctor is often limited, avoiding mentioning leprosy can simplify the diagnosis. Such experiences highlight a distinctive form of stigma construction in Colombia, where the considerations and strategies employed by patients to navigate the healthcare system differ. Recognizing and comprehending this unique conceptualization of stigma is essential for designing effective interventions that are tailored to the specific challenges faced by patients in Colombia.

When examining patients’ experiences, a significant emphasis emerged on mental health and the disability subsidy. These factors were recurrent and interconnected themes that appeared across different categories during our analysis. Despite the well-documented and measured impact of leprosy on mental well-being, it is notable that mental health considerations are often not included in the broader assessment of the disease’s burden [33]. Our findings aligned with previous studies [6] that highlighted a substantial psychosocial burden experienced by leprosy patients. In the region of Norte de Santander, this aspect appears to be overlooked, as many participants described a reality where patients struggle with depression and anxiety without sufficient support. Access to adequate mental health resources is impeded by barriers such as the cost of medicines and professional help. A multitude of factors intertwine with patients’ mental health: the intricate and lengthy leprosy journey, the treatment’s secondary effects, economic and social instability, personal fulfilment, and the experience of social isolation. Acknowledging and addressing these mental health challenges is vital for providing comprehensive care and support to leprosy patients in Colombia.

The disability subsidy is an additional intersectional factor that positively impacts a patient’s mental well-being by providing a sense of security and stability. However, the process of obtaining, managing, and renewing the subsidy poses challenges and conflicts. Firstly, eligibility for the subsidy is determined by predefined fixed categories such as the level of disability and income, which oversimplify the patient’s realities. Therefore, cases that should be considered for support are overlooked, such as the case of a patient suffering from domestic violence and depending on her aggressor for survival. Secondly, the patient’s subsidy has significant implications for their family’s financial situation which, when not monitored, places the patient in a vulnerable position. Implementing follow-up programs, financial education, and assistance in managing the subsidy is crucial to prevent exploitation and ensure its intended benefits. Thirdly, the government’s requirement of proving the patient’s survival to maintain the subsidy highlights how the bureaucratic environment can complicate matters. In response, the patients’ association has taken on the role of assisting patients with this process. Unfortunately, this arrangement obligates patients to contribute financially to the association, which can create an additional financial burden for them. Addressing these complexities and challenges associated with the subsidy requires a holistic approach that considers the diverse circumstances of patients and seeks to provide meaningful support without further burdening them.

The absence of incentives to involve expert patients as social workers stands in complete contrast to historical approaches to leprosy management, leaving a significant gap in the understanding and management of the disease. These individuals possess invaluable insights derived from their firsthand experience of living with leprosy. Their perspective could be harnessed for activities such as case detection, follow-up procedures, and information campaigns. They possess an intimate understanding of the emotional and practical challenges faced by patients –insights that are often better understood by those who have lived through them. Their unique perspectives extend to aspects such as the psychological impact of growing up with leprosy, how it shapes personality, and the day-to-day barriers that patients encounter.

The integration of alternative narratives with the biomedical framework is insufficient, highlighting the need for a stronger dialogue merging these paradigms. Bridging this gap between biomedical approaches and patient experiences is crucial for a holistic understanding of leprosy beyond historical contexts. This integration can lead to more comprehensive disease management strategies, designed with sensitivity to the multifaceted challenges of patients.

Effective efforts to combat leprosy-related stigma require a comprehensive approach that considers the broader societal context and the intricate interplay of various factors. Incorporating the experiences and voices of those affected by leprosy, as well as addressing the structural inequalities that contribute to stigma, is essential. By taking a holistic view and working towards dismantling the structural barriers that sustain stigma, it becomes possible to create more inclusive and effective strategies that genuinely support leprosy patients and their well-being.

### Limitations

This study holds particular significance given the endemic nature of leprosy in the researched area. However, there is still a need to extend the geographic scope of this study in order to fully incorporate the complexity and diversity of Colombia. It is worth noting that the study’s reach was constrained by national borders with Venezuela. Nonetheless, it is important to recognize that diseases disregard borders, warranting deeper consideration of the surrounding areas of Norte de Santander. Those regions can act as significant crossroads for disease transmission due to movements across borders. Furthermore, examining these regions emphasizes the importance of collaborative efforts across borders to manage disease control effectively.

## 5. Conclusion

This research extends the focus of stigma related to leprosy to reveal structural elements of stigma in the Colombian healthcare system. It highlights how the neglect of leprosy is evident in the organization, resource allocation, attention given, and challenges faced by healthcare workers. In conclusion, the commercialization of health, bacteriological reductionism, omission of patient narratives, and overlooking various challenges and intersectional factors throughout the leprosy journey are all clear indicators of stigma and discrimination ingrained at structural levels. This systemic bias permeates all levels of Colombian society. Failing to recognize and address this reality when crafting interventions, treatments, policies, or research perpetuates and reinforces the existing stigma surrounding leprosy.

Recommendations include involving patients in program planning, integrating clinical history systems, proactive case detection, context-specific treatment, and patient participation in public initiatives. These measures aim to empower patients, improve mental well-being, and reduce stigma within Colombian society and institutions.

## Supporting information

S1 Table Interview guide.
(PDF)

S2 Table Table of themes with their corresponding categories and main codes.
(PDF)

S1 Text Spanish version of the manuscript.
(DOCX)

S1 Checklist Inclusivity in global research questionaire.
(PDF)

## References

[1] World Health Organization. Leprosy [Internet]. 2023 [cited 2023 Jan 27]. Available from: https://www.who.int/news-room/fact-sheets/detail/leprosy

[2] White C, Franco-Paredes C. Leprosy in the 21st century. Clin Microbiol Rev. 2015;28(1):80–94. doi: 10.1128/CMR.00079-13 25567223 PMC4284303

[3] Dharmawan Y, Fuady A, Korfage IJ, Richardus JH. Delayed detection of leprosy cases: A systematic review of healthcare-related factors. PLoS Negl Trop Dis. 2022;16(9):e0010756. doi: 10.1371/journal.pntd.0010756 36067195 PMC9481154

[4] Cardona-Castro N. Leprosy in Colombia: post elimination stage?. Lepr Rev. 2013;84(3):238–47. doi: 10.47276/lr.84.3.238 24428118

[5] Gómez LJ, van Wijk R, van Selm L, Rivera A, Barbosa MC, Parisi S, et al. Stigma, participation restriction and mental distress in patients affected by leprosy, cutaneous leishmaniasis and Chagas disease: a pilot study in two co-endemic regions of eastern Colombia. Trans R Soc Trop Med Hyg. 2020;114(7):476–82. doi: 10.1093/trstmh/trz132 32052043 PMC7334822

[6] van Wijk R, van Selm L, Barbosa MC, van Brakel WH, Waltz M, Philipp Puchner K. Psychosocial burden of neglected tropical diseases in eastern Colombia: an explorative qualitative study in persons affected by leprosy, cutaneous leishmaniasis and Chagas disease. Glob Ment Health (Camb). 2021;8:e21. doi: 10.1017/gmh.2021.18 34249368 PMC8246647

[7] Dako-Gyeke M, Asampong E, Oduro R. Stigmatisation and discrimination: Experiences of people affected by leprosy in Southern Ghana. Lepr Rev. 2017;88(1):58–74. doi: 10.47276/lr.88.1.58 30188092

[8] Rai SS, Peters RMH, Syurina EV, Irwanto I, Naniche D, Zweekhorst MBM. Intersectionality and health-related stigma: insights from experiences of people living with stigmatized health conditions in Indonesia. Int J Equity Health. 2020;19(1):206. doi: 10.1186/s12939-020-01318-w 33176809 PMC7661268

[9] van Brakel WH, Cataldo J, Grover S, Kohrt BA, Nyblade L, Stockton M, et al. Out of the silos: identifying cross-cutting features of health-related stigma to advance measurement and intervention. BMC Med. 2019;17(1):13. doi: 10.1186/s12916-018-1245-x 30764817 PMC6376667

[10] Gómez L, Rivera A, Vidal Y, Bilbao J, Kasang C, Parisi S, et al. Factors associated with the delay of diagnosis of leprosy in north-eastern Colombia: a quantitative analysis. Trop Med Int Health. 2018;23(2):193–8. doi: 10.1111/tmi.13023 29230912

[11] Staples J. Nuancing ‘leprosy stigma’ through ethnographic biography in South India. Lepr Rev. 2011;82(2):109–23. doi: 10.47276/lr.82.2.109 21888136

[12] Weiss MG, Ramakrishna J, Somma D. Health-related stigma: rethinking concepts and interventions. Psychol Health Med. 2006;11(3):277–87. doi: 10.1080/13548500600595053 17130065

[13] Bijker W. Understanding technological culture through a constructivist view of science, technology, and society. In: Cutcliffe SH, Mitcham C, editors. Visions of STS; counterpoints in Science, Technology, and Society Studies. New York: State University of New York Press; 2001.

[14] Obregón D. The Social Construction of Leprosy in Colombia, 1884-1939. Science, Technology and Society. 1996;1(1):1–23. doi: 10.1177/097172189600100102

[15] Bronfenbrenner U. Ecological models of human development. In: International encyclopedia of education. Oxford, England: Elsevier; 1994.

[16] Thorogood N, Green J. Qualitative Methods for Health Research. Sage; 2018.

[17] Saldana J. The Coding Manual for Qualitative Researchers (2nd ed.). London: Sage; 2013.

[18] Tolley E, Ulin P, Mack N, Robinson E, Succop S. Qualitative methods in public health: a field guide for applied research (Second, Ser. Jossey-bass public health). Wiley; 2016.

[19] Deps P, Antunes JMA de P, Collin SM. Zoonotic risk of Hansen’s disease from community contact with wild armadillos: A systematic review and meta-analysis. Zoonoses Public Health. 2021;68(2):153–64. doi: 10.1111/zph.12783 33226194

[20] Abadía-Barrero C. Políticas y sujetos del sida en Brasil y Colombia. Rev Colomb Antropol. 2004;40:123–54.

[21] Ahumada C. La penuria de la salud pública. Rev Gerenc Polit Salud. n.d.;47 47–56.

[22] Obregón D. Building national medicine: leprosy and power in Colombia, 1870-1910. Soc Hist Med. 2002;15(1):89–108. doi: 10.1093/shm/15.1.89 12622116

[23] Laurell AC, Arellano OL. Market commodities and poor relief: the world bank proposal for health. Int J Health Serv. 1996;26(1):1–18. doi: 10.2190/PBX9-N89E-4QFE-046V 8932599

[24] Romero-Montoya M, Beltran-Alzate JC, Cardona-Castro N. Evaluation and Monitoring of Mycobacterium leprae Transmission in Household Contacts of Patients with Hansen’s Disease in Colombia. PLoS Negl Trop Dis. 2017;11(1):e0005325. doi: 10.1371/journal.pntd.0005325 28114411 PMC5289623

[25] Trapence G, Collins C, Avrett S, Carr R, Sanchez H, Ayala G, et al. From personal survival to public health: community leadership by men who have sex with men in the response to HIV. Lancet. 2012;380(9839):400–10. doi: 10.1016/S0140-6736(12)60834-4 22819662 PMC3805044

[26] Stangl AL, Earnshaw VA, Logie CH, van Brakel W, C Simbayi L, Barré I, et al. The Health Stigma and Discrimination Framework: a global, crosscutting framework to inform research, intervention development, and policy on health-related stigmas. BMC Med. 2019;17(1):31. doi: 10.1186/s12916-019-1271-3 30764826 PMC6376797

[27] Kippax S, Stephenson N, Parker RG, Aggleton P. Between individual agency and structure in HIV prevention: understanding the middle ground of social practice. Am J Public Health. 2013;103(8):1367–75. doi: 10.2105/AJPH.2013.301301 23763397 PMC4007890

[28] Platarrueda Vanegas C. La voz del proscrito o la exclusión desde adentro: lepra y representaciones sociales de los lazaretos en Colombia. Una aproximación antropológica [Doctoral thesis, Universidad Nacional de Colombia]. 2007. Available from: https://www.humanas.unal.edu.co/2017/investigacion/application/files/2115/5665/4188/Pre-La_voz_del_proscrito._Experiencia_de_la_lepra_y_devenir_de_los_lazaretos_en_Colombia.pdf

[29] Balagon MVF, Gelber RH, Abalos RM, Cellona RV. Reactions following completion of 1 and 2 year multidrug therapy (MDT). Am J Trop Med Hyg. 2010;83(3):637–44. doi: 10.4269/ajtmh.2010.09-0586 20810832 PMC2929063

[30] White C. Sociocultural considerations in the treatment of leprosy in Rio de Janeiro, Brazil. Lepr Rev. 2002;73(4):356–65. 12549843

[31] Botero Jaramillo N, Polo Rivas D, Sinuco Rueda L. La lepra en Colombia: estigma, identidad y resistencia en los siglos XX y XXI. Rev Sal Bosq. 2015;5(1):67. doi: 10.18270/rsb.v5i1.185

[32] Brewis A, Wutich A. Lazy, crazy, and disgusting: stigma and the undoing of global health. Baltimore: Johns Hopkins University Press; 2019.

[33] Somar P, Waltz MM, van Brakel WH. The impact of leprosy on the mental wellbeing of leprosy-affected persons and their family members - a systematic review. Glob Ment Health (Camb). 2020;7:e15. doi: 10.1017/gmh.2020.3 32742673 PMC7379324

