# Supplementary material for: Exploring the experiences of leprosy stigma among patients and healthcare workers in Norte de Santander, Colombia
Source: PLOS Glob Public Health. 2025 Mar 18;5(3):e0003939. doi: 10.1371/journal.pgph.0003939 (PMC11918372; doi:10.1371/journal.pgph.0003939)
Supplement: S1 Table — Interview guide. (PDF) [file pgph.0003939.s001.pdf]

**PATIENT Questions** in chronological order, request examples and reasoning for answers	**Topics/Concepts**	**Notes**
Personal introduction<br>Explanation of the study and consent<br>Where are you from? Where do you live? What do you do?<br>What is a normal day like for you?<br>What is a weekend day like for you?<br>When did you discover you had leprosy? How did you realize it was that disease?<br>Did you know anyone else with leprosy before this?<br>What happened afterward? Did anything change in your life?<br>How was the diagnostic and treatment?<br>How did you cope with the treatment process?<br>What challenges do you face every day?<br>Did you receive help from anyone?<br>How is your life now after treatment? Have you experienced any changes in your life?<br>What do you think about leprosy? What does people say? Experience, transmission, origins, misconceptions, judgments, ...<br>What does your family think about leprosy?<br>What do people think?<br>What does the doctor think?	**Anticipated/Perceived Stigma**<br>• Changes in social behavior<br>• Changes in daily life<br>**Internalized Stigma**<br>• Guilt<br>• Regret<br>• Shame<br>• Fear<br>• Self-esteem/Dignity<br>**Experienced Stigma**<br>• Physical contact<br>• Discrimination<br>• Isolation<br>• Jokes/abuse<br>• Fear<br>• Judgments<br>**Impact on Life**<br>• Respect<br>• Physical appearance<br>• Moral<br>• Endemic areas<br>**Myths and Misconceptions**<br>• Transmission<br>• Origin of the disease<br>• Religion<br>**Levels**<br>• Individual<br>• Family/Friends<br>• Community<br>• Structural	**Mentioned...** (Why or why not?)<br>Family, spouse, or partner?<br>Job?<br>Friends?<br>Leisure activities with family or friends?<br>Symptoms or doctor?<br>Have you told close contacts?<br>Any support?<br>Community isolation?<br>Job changes?<br>Shame, guilt, regret?<br>If not mentioned enough, ask why.

**DOCTOR**. **Questions**	**Topics/Concepts**	**Notes**
Personal introduction - Explanation of the study and consent<br>Where are you from? Where do you live?<br>What is a normal day like for you?<br>What is your experience in diagnosing leprosy?<br>How does the patient react when diagnosed?<br>What is your experience with the treatment process?<br>What challenges do you face every day?<br>What do you think is the priority for addressing leprosy in Colombia?<br>What do you (or your colleagues) think about leprosy? Experience, transmission, origins, misconceptions, judgments, ...	Patient Perception<br>Perception of the disease<br>Experiences of discrimination<br>Level of contagiousness<br>Guilt<br>Judgment<br>Misconceptions<br>Endemicity<br>Challenges/Barriers	**Mentioned...** (Why or why not?)<br>Family or friends?<br>Coworkers?<br>Discrimination?<br>Endemic areas?<br>Solutions?<br>Challenges?<br>Do you consider it a public health problem?

S1 Table. Interview guide.
