# Supplementary material for: Exploring the experiences of leprosy stigma among patients and healthcare workers in Norte de Santander, Colombia
Source: PLOS Glob Public Health. 2025 Mar 18;5(3):e0003939. doi: 10.1371/journal.pgph.0003939 (PMC11918372; doi:10.1371/journal.pgph.0003939)
Supplement: S2 Table — Table of themes with their corresponding categories and main codes. (PDF) [file pgph.0003939.s002.pdf]

Theme	Category	Code
Anticipated stigma	Avoid talking about it	• Keep it secret in the neighbourhood<br>• Do not tell family
	Public Image	• Collect drugs in different city<br>• Avoid other patients
	Fake excuses to symptoms	• Fungus<br>• Allergy<br>• Sunburned
Self-stigma	Euphemism	• Little thing<br>• Little spot<br>• Hansen
	Self-isolation	• Stop working<br>• Autonomous jobs
	Social life	• No friends<br>• Stay home
	Hide	• Body<br>• Leprosy documents
Experienced stigma	Discrimination	• No job<br>• Doctor distance<br>• No kiss<br>• Different cutlery
Structural discrimination	Ignorance healthcare workers	• University<br>• No aware
	Difficulties patients	• Collect treatment<br>• Subsidy<br>• Secondary effects treatment<br>• Mental impact
	Government	• Minimum funds<br>• Less time invested
	Association	• Diminish political influence<br>• Unfair charge<br>• Obligation
	Health system	• System reforms<br>• Waiting times<br>• Temporal contracts<br>• No follow up<br>• Subsidy<br>• IDS
	Barriers	• Better organization before<br>• Challenges HW<br>• Migration<br>• Big and separate families<br>• Late diagnosis<br>• Ineffective training<br>• Red Zones

**Theme**	**Category**	• **Code**
Misconceptions and beliefs	Patients	• Uneducated<br>• Bad lifestyles and conditions
	Origen	• Hereditary<br>• Black magic<br>• God's plan<br>• Armadillo<br>• Working with coal<br>• Prison
	Transmission	• Fast and far
	Fear	• Consequences<br>• Origen<br>• Family
Local characteristics	Refuse doctors	• Fear<br>• No trust
	Alternative knowledge	• Natural remedies<br>• Experienced patients<br>• Traditional healers
	Religion	• Origen<br>• Treatment<br>• Reasoning
	Leprosariums	• Old stories

S2 Table. Table of themes with their corresponding categories and main codes.
