# Supplementary material for: Exploring the experiences of leprosy stigma among patients and healthcare workers in Norte de Santander, Colombia
Source: PLOS Glob Public Health. 2025 Mar 18;5(3):e0003939. doi: 10.1371/journal.pgph.0003939 (PMC11918372; doi:10.1371/journal.pgph.0003939)
Supplement: S1 Text — Spanish version of the manuscript. (DOCX) [file pgph.0003939.s003.docx]

Explorando las experiencias del estigma de la lepra entre pacientes y trabajadores de la salud en Norte de Santander, Colombia

Título corto: El estigma de la lepra en Colombia

Carlos Mayoral-García1,3, Anil Fastenau1,2,3, Cristian Ghergu1

^1^ Facultad de Salud, Medicina y Ciencias de la Vida, Universidad de Maastricht, Maastricht, Países Bajos

^2^ Asociación Alemana de Ayuda a la Lepra y la Tuberculosis (GLRA/DAHW), SEDE, Würzburg, Alemania

^3^ Asociación Alemana de Ayuda a la Lepra y la Tuberculosis (GLRA/DAHW), región América del Sur, Bogotá, Colombia

**Autor para correspondencia**

Carlos Mayoral-García - mayoralgarciacarlos@gmail.com

Anil Fastenau - anil.fastenau@dahw.de

# Abstracto

**Introducción:** Este estudio examina el estigma relacionado con la lepra entre pacientes y profesionales de la salud en Colombia. La lepra, clasificada como una ETD incluida en la lista de la OMS, es una enfermedad nerviosa crónica que causa pérdida sensorial, discapacidades y deformidades cuando no se trata. Esto contribuye al estigma, reduciendo la calidad de vida, el acceso a la atención médica y los ingresos. A pesar de que Colombia ha alcanzado el objetivo de prevalencia de la OMS, algunas regiones aún enfrentan altas tasas de detección. Como resultado, la lepra sigue siendo un desafío debido a una comprensión incompleta de la carga completa de la enfermedad y sus factores entrelazados.

**Metodología:** El estudio constó de 25 entrevistas a pacientes y trabajadores de la salud, y visitas de campo en el departamento colombiano de Norte de Santander. Empleando un enfoque constructivista para contextualizar la lepra en Colombia a través de factores históricos y socioeconómicos, integramos las perspectivas de los participantes para permitir flexibilidad más allá de las rígidas categorías de estigma de la psicología y el enfoque estrecho de la enfermedad.

**Resultados:** Los hallazgos de nuestra investigación confirman la investigación regional sobre la estigmatización de los pacientes, incluido el estigma anticipado, interno y experimentado, con un enfoque particular en el nivel estructural y los factores interseccionales. Este estigma se hace evidente cuando se examina la organización del sistema de salud, la asignación de recursos para la prevención, el diagnóstico y el tratamiento de la lepra, y la atención inadecuada a la salud mental de los pacientes. Además, describimos la comercialización de la salud en Colombia, que perpetúa esta situación al socavar la red comunitaria de lepra previamente establecida, reducir la enfermedad a una mera perspectiva bacteriológica y silenciar las narrativas de los pacientes.

**Conclusión:** Nuestra investigación proporciona información valiosa para mejorar la detección, el diagnóstico, el tratamiento y la inclusión social de los casos de lepra y, en última instancia, mejorar la calidad de vida de los pacientes. Las recomendaciones para las políticas de salud pública de Colombia incluyen la participación de los pacientes en los programas de lepra, la mejora de los sistemas nacionales de historia clínica, la implementación de la detección activa de casos, la adaptación de los tratamientos a los contextos locales y el fomento de la participación de los pacientes en iniciativas públicas integrales. Estas medidas empoderan a los pacientes, impactan positivamente en su bienestar mental y combaten el estigma arraigado en la sociedad y las instituciones colombianas.

**Palabras clave:** Lepra, investigación cualitativa, estigma, Colombia

# 1. Introducción

La lepra es una enfermedad infecciosa crónica clasificada por la Organización Mundial de la Salud (OMS) como una de las veinte Enfermedades Tropicales Desatendidas (ETD) y es causada por una bacteria llamada *Mycobacterium leprae* que afecta los nervios periféricos, la piel, los ojos y las membranas mucosas [1]. La presencia de los bacilos dentro de las células de la piel causa manifestaciones dermatológicas y, cuando no se trata, la infección de los nervios conduce a la pérdida sensorial, discapacidad y deformidad [2]. En muchos casos, el diagnóstico llega tarde debido al estigma, la falta de conciencia o los problemas relacionados con la atención médica que contribuyen a las deficiencias físicas, lo que plantea un desafío importante para la rehabilitación del paciente [3].

Hace dos décadas, Colombia alcanzó la meta de prevalencia de la OMS de 1 caso por cada 10.000 habitantes, criterio para dejar de considerar a la lepra como un problema de salud pública. Sin embargo, ciertas regiones colombianas se han mantenido endémicas, con Arauca y Norte de Santander exhibiendo tasas de detección de 4,73/10.000 y 3,86/10.000 respectivamente [4]. El modesto número de casos nacionales, que ascendió a 272 en 2022 [1], contribuye a la financiación pública inadecuada, la investigación limitada, la escasez de expertos y la insuficiencia de instalaciones sanitarias. En consecuencia, persiste un problema prevalente de diagnóstico tardío, lo que lleva a que el 30% de los nuevos casos manifiesten discapacidades y el 10% experimente discapacidades de grado 2. En el contexto de las zonas endémicas dentro de Colombia, se han realizado dos estudios. El primero, realizado por Gomez et al. en 2020 [5], evaluó el estigma asociado con la lepra, descubriendo que el 49% de los participantes experimentó angustia mental y el 27% enfrentó restricciones de participación. Explorando más a fondo las dimensiones del estigma prevalente, otro estudio realizado en las mismas regiones reveló que cerca del 70% de los pacientes con lepra están lidiando con un estigma sustancial anticipado y experimentado. Esto afecta negativamente a su bienestar psicológico y dificulta su integración social, exacerbando así la carga general que supone la enfermedad. El estigma de la lepra se observa a menudo en los círculos familiares, de amigos y vecinos [6]. Sin embargo, estudios realizados en Colombia y otros países [6-8], indican que los prestadores de salud también contribuyen a la estigmatización de los pacientes.

Con esta investigación, queremos obtener información cualitativa sobre el estigma de la lepra con un enfoque crítico en los contextos socioculturales, históricos, económicos y políticos en los que se produce el estigma. A través de esta exploración cualitativa, aspiramos a develar caminos alternativos que diverjan de las normas académicas convencionales, como se demuestra en el trabajo de van Brakel et al. [9]. Su enfoque aboga por comprender el estigma relacionado con la salud como un constructo universal, aplicable a diversas culturas y condiciones de salud. Esta postura facilita la aplicación de un concepto de estigma estandarizado, lo que permite la comparación de datos y fomenta la colaboración mundial. Sin embargo, si bien este enfoque simplifica el análisis de datos y la cooperación internacional, también simplifica en exceso la compleja cuestión y su evaluación a menudo se limita a parámetros estrechos, empleando herramientas y escalas estandarizadas que no abarcan la totalidad del impacto de la enfermedad.

Se han empleado métodos similares para dilucidar el estigma en nuestra área de investigación [10]. Sin embargo, este enfoque no proporciona una visión completa del estigma, ya que se limita principalmente a la descripción y la medición en lugar de ofrecer una comprensión holística. Otro estudio realizado en Colombia [6] empleó grupos focales y entrevistas semiestructuradas, aunque su análisis y hallazgos permanecieron confinados dentro del marco general del estigma conceptualizado por van Brakel et al. [9]. A la luz de esta situación, surge una notable brecha de investigación dentro de la literatura existente, lo que pone de relieve la necesidad de mejorar nuestra comprensión del estigma en esta región específica. Muchos estudios sobre el estigma de la lepra subestiman las identidades sociales interseccionales como la casta, la clase y el género, lo que a menudo simplifica las experiencias sociales complejas, lo que refuerza la necesidad de un enfoque etnográfico más matizado para abordar plenamente el problema [11].

Solo al obtener una perspectiva integral de la intrincada interacción y las repercusiones de la lepra en la vida de los pacientes, podemos elaborar intervenciones efectivas para la erradicación del estigma y la recuperación completa de los pacientes. Por lo tanto, es imperativo desafiar la conceptualización dominante del estigma que se encuentra en la literatura. Esto implica tener en cuenta los factores locales y evadir una comprensión aislada del estigma.

El objetivo principal de nuestro estudio fue investigar el estigma relacionado con la lepra entre los participantes. Una vez identificado, nuestro objetivo fue comprender cómo se experimenta este estigma tanto por parte de los pacientes como por los profesionales de la salud. Al explorar el estigma relacionado con la salud, pretendemos recopilar información para informar futuras intervenciones en el país. Por lo tanto, la pregunta de investigación que guió este estudio es la siguiente:

*¿Cómo se vive el estigma de la lepra en las zonas endémicas de Colombia?*

Las subpreguntas incluyen:

- *¿Cómo experimentan los pacientes el estigma de la lepra?*
- *¿Cómo viven los trabajadores de la salud el estigma de la lepra?*
- *¿Qué contextos sociales, políticos y económicos contribuyen al estigma?*

# 4. Metodología

## 4.1. Diseño del estudio

La definición de estigma utilizada en este estudio es, como afirman Weiss et al., "un proceso social o experiencia personal relacionada caracterizada por la exclusión, el rechazo, la culpa o la devaluación que resulta de la experiencia o la anticipación razonable de un juicio social adverso sobre una persona o grupo identificado con un problema particular" [12].

La fundamentación teórica fundamental de esta investigación parte de una perspectiva constructivista de la ciencia y la tecnología, articulada por Bijker [13], y se inspira en el trabajo seminal de Diana Obregón [14] sobre la construcción social de la lepra en Colombia. Al adoptar esta perspectiva, aspiramos a diseccionar las intrincadas dimensiones que subyacen a la construcción del estigma, develando la compleja red de dinámicas que influyen en su evolución dentro del contexto de la lepra.

A través de la perspectiva constructivista, utilizamos dos modelos ampliamente adoptados en la academia debido a su aplicabilidad y facilidad de uso: el Modelo Socio-Ecológico [15] y el Modelo de Estigma Relacionado con la Salud [9]. Al aplicar el Modelo de Estigma Relacionado con la Salud, desarrollamos temas en torno al estigma anticipado, el autoestigma y el estigma experimentado, lo que proporcionó un marco claro para explorar las dinámicas individuales e interpersonales del estigma en las personas afectadas por la lepra.

Para ampliar el alcance de este análisis, incorporamos el Modelo Socio-Ecológico, lo que nos permite contextualizar el estigma dentro de estructuras sociales más amplias. Este modelo agregó una capa de macrosistema que capturó las influencias más grandes sobre el estigma, abordando específicamente la discriminación estructural, los conceptos y creencias erróneos, y las características locales de la región. Estos temas adicionales nos permitieron examinar cómo los factores sociales y ambientales dan forma a la percepción y la experiencia del estigma en este contexto particular. En la Figura 2 a continuación, podemos ver cómo están interconectados ambos modelos para servir a nuestros propósitos analíticos.

Figura 2. Modelo Socio-Ecológico de Estigma Relacionado con la Salud

Nuestro enfoque constructivista implica una interpretación dinámica de este conocimiento y nos anima a comprometernos con estos modelos activamente en lugar de adoptarlos pasivamente. Enraizados en estos principios, contextualizamos estos modelos dentro de las realidades prácticas, reconociéndolos como constructos que ofrecen utilidad para guiar a los individuos a través de sus entornos.

## 4.2. Entorno del estudio y selección de participantes

El estudio se llevó a cabo en el departamento de Norte de Santander, en el norte de Colombia, región seleccionada por ser considerada un área endémica con una tasa de detección de 3,86/10.000 [4]. Entre las veinticinco entrevistas realizadas, veinte participantes habían completado su tratamiento, lo que les permitió proporcionar un relato exhaustivo de su experiencia con la enfermedad. Además, dos de ellos participaron activamente como trabajadores sociales en campañas contra la lepra. Reconociendo la investigación previa [7,8] que identificó la estigmatización por parte de los proveedores de atención médica, incluimos una pequeña muestra de trabajadores de la salud para obtener información sobre las prácticas diarias de atención al paciente: se realizaron cinco entrevistas con profesionales de la salud experimentados, incluida una enfermera jefe de un centro de salud, dos dermatólogos, un trabajador social y un coordinador de lepra. La mayoría de los participantes tenían más de 40 años, con 17 mujeres y 8 hombres. Las entrevistas con los pacientes se llevaron a cabo dentro de sus residencias, mientras que las de los trabajadores de la salud se llevaron a cabo en habitaciones privadas.

## 4.3. Recopilación de datos

Los participantes fueron seleccionados a través de un muestreo intencional, abarcando exclusivamente individuos adultos (mayores de 18 años) residentes en el área de estudio, pacientes con diagnóstico confirmado de lepra y trabajadores de la salud. La estrategia de muestreo buscó la heterogeneidad, abarcando una amplia gama de individuos de diversos orígenes para garantizar una comprensión integral de las experiencias vividas. Para lograr esta diversidad, se reclutó a los participantes con la asistencia de trabajadores sociales del Instituto de Salud de Norte de Santander, considerando su disposición y disponibilidad. El período de reclutamiento fue del 18 de mayo de 2023 al 3 de julio de 2023, y todos los participantes dieron su consentimiento informado por escrito.

Las entrevistas fueron realizadas personalmente por el investigador principal en español, su lengua materna. Sin embargo, para establecer la confianza y cultivar una fuerte conexión entre los participantes y el investigador, un trabajador social local experimentado y bien considerado dentro de la comunidad facilitó las presentaciones.

Las entrevistas tuvieron una duración aproximada de 30 a 90 minutos y fueron grabadas en formato de audio. Después de la introducción y la obtención del consentimiento informado, la entrevista comenzó bajo una guía preconstruida (Tabla S1) que contenía preguntas abiertas, alentando a los participantes a proporcionar respuestas detalladas. En respuesta a las ideas emergentes, se aplicaron revisiones a las guías temáticas en consecuencia. El número de entrevistas se determinó con base en los objetivos de la investigación y la disponibilidad de participantes dentro de la población de pacientes con lepra. La recopilación de datos continuó hasta que quedó claro que no estaban surgiendo nuevos temas o ideas, lo que indicaba que se había alcanzado la saturación de datos. Garantizamos la confiabilidad explorando las perspectivas y experiencias de múltiples actores: pacientes, trabajadores de la salud y trabajadores sociales. Además, la confirmabilidad se mantuvo a través de la reflexividad, con el investigador reflexionando continuamente sobre los posibles sesgos y buscando retroalimentación de sus pares y colaboradores locales para garantizar la objetividad.

## 4.4. Análisis de datos

Después de transcribir las entrevistas y familiarizarnos con los datos, empleamos el análisis de contenido temático [16] para organizar y sintetizar los temas emergentes. El marco teórico que adoptamos sirvió como punto de partida, sin embargo, abordamos los datos con una perspectiva constructivista, permitiendo que los temas, categorías y códigos emergieran de manera deductiva, lo que condujo a datos más ricos y una comprensión más amplia. Si bien los enfoques deductivos a menudo se basan en temas predefinidos, reconocimos la necesidad de una codificación inductiva para capturar los matices de las experiencias de los participantes, lo que facilita un análisis más completo de los datos.

Para cada tema, desarrollamos categorías y códigos detallados [17]. Los códigos se refinaron para mayor especificidad y para evitar la repetición. Las categorías se cruzaron con temas codificados por colores, alineándose tanto con nuestro marco teórico como con los temas que surgieron de las entrevistas. La lista completa de categorías bajo cada tema, junto con los códigos principales, se puede encontrar como una tabla en la información de soporte (Tabla S2). A lo largo de la recopilación y el análisis de datos, el investigador se mantuvo consciente de sus ideas preconcebidas, su contexto social y sus influencias más amplias sobre los datos.

## 4.5. Consideraciones éticas

Dada la naturaleza sensible de la enfermedad bajo investigación, pusimos especial énfasis en garantizar la participación voluntaria y abordar las preocupaciones relacionadas con la coerción [18]. Para garantizar la comodidad y mantener la confidencialidad, se dio a los participantes la libertad de seleccionar un entorno privado para las entrevistas. Además, se explicaron de manera exhaustiva los objetivos del estudio y las medidas de privacidad, lo que brindó a los participantes la oportunidad de buscar aclaraciones y hacer preguntas. Todos los participantes dieron su consentimiento informado por escrito. Al realizar una investigación en Colombia, reconocemos el contexto colonial histórico y las implicaciones de la presencia de un investigador español. Por lo tanto, nuestro estudio incorpora consideraciones específicas, como la sensibilidad cultural, la colaboración con expertos locales, el respeto a la autonomía de los participantes, la reciprocidad, la transparencia, la reflexión continua sobre la posición de los investigadores y el diálogo abierto para abordar las preocupaciones éticas. Estas prácticas garantizan un proceso de investigación ético y respetuoso. Se obtuvo la aprobación ética de los comités de ética tanto de la Universidad de Maastricht en los Países Bajos como de la Universidad de Santander en Cúcuta, Colombia, número de registro CEI-ISEM-02-2023.

# 5. Resultados

Este estudio cualitativo incluye 25 entrevistas semiestructuradas realizadas de mayo a julio de 2023 en la región de Norte de Santander, Colombia. Empleando el modelo conceptual de estigma propuesto por van Brakel et al. [9], pudimos construir sobre los hallazgos presentados por van Wijk et al. [6], particularmente en relación con la presencia de diversas formas de estigma dentro del contexto colombiano. La prevalencia del estigma anticipado fue evidente entre nuestros participantes, quienes se abstuvieron de divulgar su condición a familiares y conocidos, a menudo recurriendo a excusas u ocultando su enfermedad a través de diversos medios. Por otro lado, el estigma internalizado se encontró con menos frecuencia, ya que muchas personas poseían una comprensión integral de la lepra. No obstante, un sentimiento prevalente entre los participantes fue un debilitamiento de la seguridad en sí mismos y un sentimiento de disminución de la valía debido a las limitaciones sociales y laborales provocadas por la enfermedad. Por lo tanto, la experiencia del estigma se desencadenó predominantemente cuando los pacientes eligieron compartir su diagnóstico con otros. Si bien los miembros de la familia tendían a brindar apoyo, los casos de discriminación solían surgir de la comunidad y de los trabajadores de la salud. Sin embargo, como exploraremos más adelante, es importante reconocer que una fuente sustancial de discriminación experimentada se deriva de un nivel estructural, interrelacionado con la forma en que se organiza el sistema de salud y la percepción predominante de la enfermedad.

Las entrevistas arrojan luz sobre la estigmatización y el abandono que conlleva la carga de la lepra, subrayando los problemas dentro de la organización del sistema de salud, la asignación de recursos para la prevención, el diagnóstico y el tratamiento, la atención prestada al bienestar mental de los pacientes y los desafíos que enfrentan los trabajadores de la salud en la práctica. Además, nuestro análisis explora las tendencias prevalentes, en particular la comercialización de la atención médica. Esta tendencia perpetúa la situación al socavar la red histórica de la comunidad de lepra, reducir la comprensión de la enfermedad a un mero punto de vista bacteriológico y silenciar las narrativas de los pacientes.

La sección de resultados se divide en tres capítulos, cada uno de los cuales aborda los temas clave identificados en el estudio. El primer capítulo, El viaje del paciente, sigue la progresión de la lepra descrita por los pacientes, desde el inicio de los síntomas hasta el diagnóstico, el tratamiento y la recuperación. Esta estructura refleja cómo se desarrollaron naturalmente las entrevistas, lo que hace que la narración sea más atractiva y fácil de seguir.

El segundo capítulo se centra en la salud mental, un tema recurrente en las entrevistas. Dada la falta de recursos de salud mental en la región y la importante carga emocional a la que se enfrentan los pacientes con lepra, nos pareció esencial dedicar un capítulo aparte a este tema.

El último capítulo examina la discriminación estructural, el tema más crítico de nuestra investigación. Explora las barreras sistémicas y los estigmas sociales que afectan a los pacientes con lepra, haciendo hincapié en su profundo impacto en sus experiencias.

## 5.1. Recorrido de los pacientes

En la sección de resultados, navegamos por el viaje tanto de los trabajadores de la salud como de los pacientes a través de los desafíos que plantea el sistema de salud colombiano. La estructura de esta sección se ha organizado en torno a las etapas clave de la enfermedad: diagnóstico, tratamiento y recuperación. Es importante tener en cuenta que, si bien presentamos este viaje de manera estructurada, la realidad está lejos de ser lineal debido a su complejidad. Por ejemplo, los pacientes pueden encontrar diagnósticos erróneos que conducen a cambios en el tratamiento o recaídas, lo que ilustra la naturaleza compleja de su experiencia. Además, este viaje no se limita a un período de tiempo o lugar específico, ya que nuestros datos incluyen información de personas que se han sometido a varios tratamientos o programas de manejo de la lepra.

Nuestro estudio compara el tratamiento de la lepra desde el pasado hasta el presente en medio de cambios sociales y políticos significativos. Los participantes que experimentaron estos cambios destacan la evolución de una comunidad sanitaria cohesionada a una estructura fragmentada, como se ve en sus referencias al apoyo proporcionado en el pasado por el IDS a lo largo de su trayectoria con la lepra.

Antes, podías ir al IDS, hacerte una baciloscopia el mismo día y, después de una semana, volver a recoger tu medicación. Todo se manejó en un solo lugar, sin tiempos de espera. (Paciente 2)

El deterioro de la asociación de pacientes

[La asociación] ha cambiado mucho porque ya no sirve (...) Vas allí, te sientas, te cobran y ya está. Eso no es una asociación. (Paciente 2)

o el sistema de salud actual dividido entre diferentes proveedores.

Los programas eran verticales porque la autoridad sanitaria gestionaba todo: vigilancia, testeos, tratamiento, seguimiento y detección activa de casos. No delegaron estas responsabilidades a diferentes proveedores de atención médica como lo hacen ahora. (HW 2)

La introducción del tratamiento con múltiples fármacos acortó la terapia, pero causó efectos secundarios como el oscurecimiento de la piel. Otro cambio fundamental fue el paso de los programas verticales, supervisados por el Instituto Departamental de Salud (IDS), que gestionaba la vigilancia, las pruebas, el diagnóstico, el tratamiento y la búsqueda activa de casos, a un sistema descentralizado. Bajo esta nueva estructura, las instituciones privadas, conocidas como Entidades Promotoras de Salud (EPS), administran servicios de salud a la población a través de una red de clínicas públicas y privadas, conocidas como Instituciones Proveedoras de Salud (IPS). Este complejo sistema funciona con las contribuciones de las personas empleadas y ofrece subsidios para los desempleados. En la actualidad, el IDS desempeña una función consultiva, garantizando el cumplimiento del protocolo. Otros actores son la debilitada pero activa asociación de pacientes y los antiguos leprosarios de Agua de Dios y Contratación, que ahora ofrecen subsidios y residencias de ancianos. Sin embargo, los desafíos contemporáneos incluyen tiempos de espera prolongados, rotación de personal, quiebras de administradores, corrupción, falta de conocimiento generalizado sobre los servicios disponibles, comercialización de la atención médica, pérdida de historial clínico, migración interna y afiliación a EPS. La descripción dada por un dermatólogo experimentado ilustra esta situación:

Es una especie de comercialización (...), ellos [las EPS] también necesitan ganar dinero. (...) Lo que pasa es que las EPS están jugando para que la gente no se enferme. (...) El médico le envía una resonancia magnética y luego le dice: "Espera, es mejor que la mire otra persona" o la retrasan. Hay tres meses de espera. Luego, cuando van a hacer la resonancia magnética, el paciente está aún más, o si ha muerto, entonces ya se ahorraron de pagar la resonancia magnética. (HW 3)

## 5.1.1 Diagnóstico

Durante nuestras entrevistas, observamos tres escenarios principales en la trayectoria de las personas con lepra, que a menudo comienza cuando notan decoloraciones inusuales de la piel. Recurren al autodiagnóstico y usan cremas de venta libre, ignoran el problema por completo o buscan una cita con un médico general.

Algunos pacientes visitan al médico, mientras que otros permanecen indiferentes y optan por no buscar ningún tratamiento. (…) Otros se desaniman por los largos tiempos de espera y optan por el autodiagnóstico, ir a una farmacia y comprar una crema. (HW1)

Cuando los pacientes consultan a un médico, el proceso de diagnóstico se vuelve complicado debido a desafíos como la atención inadecuada a la lepra, problemas de comunicación y otros factores complicados.

Hay otras enfermedades que también afectan a la piel y a los nervios que no son lepra. Por lo tanto, debes, dentro del conocimiento científico, ser capaz de diferenciarte de otras patologías. (HW 3)

Como resultado, una parte importante de nuestros participantes recibieron diagnósticos incorrectos y posteriormente se sometieron a tratamientos para diferentes enfermedades como micosis, eczema, dermatitis, psoriasis o alergias. La mayoría de ellos reciben un diagnóstico correcto cuando comienzan a experimentar una pérdida de sensibilidad en sus extremidades y se les programa una baciloscopia y una biopsia. Sin embargo, debido a las largas listas de espera, este proceso puede durar varios meses, lo que refleja la considerable demora promedio en Colombia, que se sitúa en 2,7 años [19]. Curiosamente, ni los pacientes ni los médicos anticipan un diagnóstico de lepra, ya que se percibe ampliamente como una enfermedad del pasado, que comúnmente se creía erradicada:

Cuando le digo a mi familia que tengo un paciente [de lepra], todos dicen: ¿Qué? ¿Realmente? Pensé que eso ya no existía. (HW1)

El proceso descrito es complejo y prolongado, exacerbado por la supervisión inicial de la lepra. Las múltiples citas con el médico agravan los desafíos, ya que requieren que los pacientes visiten repetidamente los centros de salud, lo que causa tensión personal y financiera. Durante este tiempo, el daño a los nervios empeora, lo que genera frustración y desconfianza en el sistema de salud. Algunos pacientes culpan a los médicos por pasar por alto la lepra, a pesar de que los antecedentes familiares lo sugieren. Tales frustraciones pueden impedir la adherencia al tratamiento y la aceptación de las visitas domiciliarias debido a la disminución de la confianza. Esta desconfianza afecta las interacciones médico-paciente, ya que los pacientes a menudo evitan hablar sobre la lepra con los médicos por temor a diagnósticos sesgados basados en su historial con la enfermedad.

No me gusta ir al médico porque, quiero decir, siempre van a relacionar todo con eso [la lepra]. Entonces, nunca lo menciono. (Paciente 13)

Se ha destacado anteriormente que el estigma es un concepto socialmente construido que adquiere diversas connotaciones en función del contexto. En este entorno particular, el estigma está moldeado por el contexto local, diferenciándose así de los asociados a otras enfermedades y situaciones. En este caso, las interpretaciones del paciente sobre la enfermedad influyen significativamente en la percepción del estigma, ya que sus explicaciones sirven de racionalización. En los casos en que la comprensión científica es incompleta o no comprensible, los pacientes construyen su sentido de comprensión. La lepra a menudo se considera como una enfermedad enigmática, lo que da lugar a explicaciones alternativas que podrían clasificarse como conceptos erróneos dentro del discurso médico:

Creo que esta enfermedad es muy misteriosa (...) porque veo y conozco a personas que nunca la contrajeron a pesar de que convivieron con personas afectadas, y luego personas que nunca conocieron a un paciente y aparecieron con la enfermedad, esto es un misterio. (Paciente 2)

Con frecuencia, los participantes compartieron experiencias de tener parientes con lepra. Sin embargo, en los casos en que los pacientes carecían de tales conexiones, establecer la causalidad fue problemático:

Digo que me contagié porque ayudaba a mi esposo, solíamos quemar carbón, entonces digo que fue eso. Tuve que sacar el carbón del fuego con un palo y luego cargarlo en un carro para venderlo y esas cosas, con productos de ese tipo (...). Sí, digo que tenía que ser el calor y eso. (Paciente 10)

Con frecuencia, los pacientes encuentran significado en la enfermedad buscando explicaciones basadas en sus propias circunstancias, asociándola con el empleo previo en condiciones peligrosas. La naturaleza oculta de la lepra contribuye a un aura de incertidumbre en torno a sus orígenes, lo que lleva a quienes no están familiarizados con la enfermedad a adoptar interpretaciones alternativas. La atribución de la lepra a vivir en entornos desfavorables y otros factores sociales altera el estigma de ser contagioso a ser indicativo de pobreza. Además, algunos participantes se esforzaron por disminuir el impacto de la enfermedad borrando los límites entre los pacientes y los individuos sanos:

No sé si es verdad o mentira, pero la gente dice que prácticamente todos tenemos la enfermedad del bacilo (...) pero simplemente se escala en uno y no en otros, entonces ¿por qué vamos a discriminar a alguien por cualquier enfermedad? (Paciente 2)

Los participantes a menudo atribuyen su infección a factores como las migraciones fronterizas con Venezuela, las vulnerabilidades inmunológicas y las prácticas dietéticas. Curiosamente, las influencias estructurales más amplias que afectan los esfuerzos de control de enfermedades en el país, como la limitada cobertura de salud y la débil aplicación de la ley en áreas remotas, o la falta de coordinación en las regiones fronterizas, a menudo no se mencionan. Estos factores entrelazados están estrechamente relacionados con el estigma y están sujetos a la configuración contextual. En particular, en línea con otros estudios [20] que indican una fuerte asociación entre el contacto directo con armadillos silvestres y un mayor riesgo de lepra, algunos participantes, incluidos pacientes, trabajadores de la salud y dermatólogos, identificaron al armadillo como un posible vector de transmisión. Si bien algunos expresaron dudas sobre su veracidad, aún lo consideraron importante para la salud pública:

La persona que come mucho armadillo contrae la enfermedad porque el armadillo tiene esa enfermedad. Los ocañañenses lo comen mucho. Y hay muchos pacientes allí. Por lo tanto, deberías decirle a la gente que no coma eso. (Paciente 2)

Otro factor es la religión, que sirve como piedra angular fundacional no solo en el contexto histórico de la lepra sino también dentro de la sociedad colombiana, jugando un papel importante en la interpretación de la enfermedad. A diferencia de ser una fuente de conflicto o discriminación, los participantes invocan con frecuencia la religión para dar sentido al origen y la progresión de la enfermedad. Incluso cuando se les pregunta acerca de sus creencias acerca de por qué Dios permite la lepra, la religión emerge como un marco a través del cual buscan comprensión:

Dios le dio las habilidades al doctor Hansen, quien descubrió los bacilos y fabricó las drogas. (…) Consiente la enfermedad con el fin de mejorar la ciencia o para tener más investigación, para que encuentren la cura. (Paciente 2)

Los trabajadores de la salud tienen sus propias perspectivas sobre la lepra, influenciadas por su mínima inclusión en su formación y los encuentros poco frecuentes a lo largo de sus carreras, lo que resulta en su eventual negligencia en la práctica. La falta de énfasis en la lepra dentro de su educación profesional contribuye a la propagación del estigma en este contexto, influyendo posteriormente en sus futuras interacciones con la enfermedad. Esto fue particularmente evidente en las explicaciones de los pacientes, donde los encuentros con médicos que mostraban aversión al tacto o luchaban por explicar la enfermedad resultaron en experiencias discriminatorias.

Algunos pacientes con lepra a largo plazo poseen un profundo conocimiento de la enfermedad, que a menudo supera al de algunos médicos. Su conocimiento abarca desde procedimientos médicos como la baciloscopia hasta regímenes de tratamiento y desarrollos históricos. Participan activamente en las discusiones médicas, ofrecen conocimientos invaluables derivados de décadas de experiencia de primera mano, contribuyendo a la detección de nuevos casos y brindando apoyo empático a otros pacientes. Su empatía, derivada de experiencias compartidas, ayuda a aliviar las aprensiones de los pacientes, especialmente en lo que respecta al estigma público. Aprovechar su experiencia no solo fomenta la inclusión, sino que también los empodera con un papel significativo en la atención al paciente.

Cuando ella [la paciente] me abrazó, me dijo: Me encanta que lo hayas hecho [la visita]. Eso es lo que me gusta, me gusta cuando hago las visitas porque a veces hay muchas preguntas, y puedo responderle al paciente todas ellas. (Paciente 2)

Este enfoque demostró ser muy eficaz en el pasado, ya que los promotores dedicados a la lepra acompañaron a los pacientes a lo largo de todo su viaje con la enfermedad. Este éxito se atribuyó a los mayores recursos asignados a los programas de lepra, a un enfoque más paternalista y a una acción rápida e integral que abarca todos los aspectos esenciales del diagnóstico, el tratamiento y la recuperación.

La ventaja era que atendían al paciente, pero al mismo tiempo los miembros de la familia y las condiciones sociales, proporcionaban tratamiento y se aseguraban de que el paciente tomara los medicamentos. Al mismo tiempo, consideraron el nivel de discapacidad y solicitaron el subsidio por discapacidad. (HW 4)

Los factores estructurales locales plantean barreras significativas para el sostenimiento de esta práctica debido a los cambios en la organización de la salud. Muchos pacientes, en su mayoría de bajos ingresos, dependen de la atención médica subsidiada, que prohíbe asumir roles como trabajadores sociales de lepra o cualquier otro trabajo a menos que dejen de ser subsidiados y comiencen a pagar el seguro médico. La ausencia de transporte oficial del IDS se suma al desincentivo, requiriendo gastos personales para los costos relacionados con el trabajo. En consecuencia, los pacientes se sienten marginados en el diseño del programa, lo que pone de relieve los factores contextuales en la formación del estigma. Su renuencia a participar se debe a obstáculos estructurales más que a un estigma previsto. Durante las visitas de campo, las personas que presentaban síntomas de lepra se acercaron a nosotros en busca de información, subrayando la necesidad de mejorar las estrategias de prevención y cuestionando las tasas de incidencia prevalecientes.

La fase de diagnóstico se destaca como uno de los aspectos más desafiantes del tratamiento de la lepra. La falta de concienciación sobre la lepra, tanto entre los médicos como entre los pacientes, junto con los resultados falsos negativos, los diagnósticos erróneos, el agotamiento de los médicos que conduce a una atención rápida e inadecuada, y la reticencia de los pacientes hacia los médicos y los enfoques biomédicos, contribuyen colectivamente al retraso en los diagnósticos. Por lo general, en el momento en que se establece el diagnóstico, los pacientes ya están experimentando una sensibilidad reducida y un daño nervioso irreversible.

## 5.1.2 Tratamiento

En Colombia, el tratamiento de la lepra es gratuito, y los pacientes deben visitar mensualmente los centros de salud para recibir medicamentos. Sin embargo, el acceso al tratamiento plantea desafíos. Muchos pacientes, tratando de evitar el estigma, prefieren la entrega a domicilio o lugares de tratamiento no revelados. Sin embargo, la entrega a domicilio no es práctica debido a los limitados recursos de IDS y a que los viajes de larga distancia imponen tensiones financieras a los pacientes, lo que a menudo provoca interrupciones en su tratamiento.

Además, en regiones remotas como el norte de Norte de Santander, particularmente cerca de Tibú, el acceso a la atención médica es aún más difícil debido a la historia de conflictos y actividades ilícitas de la zona. Designada como Zona Roja (áreas con frecuentes conflictos entre las fuerzas oficiales y los insurgentes), la entrada y salida requieren permisos, lo que aumenta la complejidad. Los residentes se enfrentan a dificultades diarias, como el desplazamiento y la pérdida de familiares debido al conflicto en curso. El estigma para los pacientes en este contexto está profundamente entrelazado con estos desafíos, lo que afecta todos los aspectos de sus vidas y la adherencia al tratamiento. La situación se ejemplifica con un incidente en el que un grupo paramilitar utilizó la fuerza para garantizar que un paciente continuara con el tratamiento:

Hace un tiempo un paciente positivo tomó la primera ampolla del tratamiento y luego dijo que no iba a continuar. ¿Saben lo que hicieron los "paracos"? Ejecutado. Le disparan. (HW 5)

La situación de inseguridad pública abarca otros factores interrelacionados, incluidos los desafíos que enfrentan los pacientes que trabajan como cultivadores de coca. Las iniciativas del gobierno y los intereses contrapuestos de varios grupos han resultado en una disminución de la demanda de coca, dejando a estos agricultores sin ingresos estables. Esto, a su vez, repercute negativamente en su capacidad para acceder al tratamiento necesario:

Así que ahora, como la coca no les da lo suficiente, ni siquiera para comer, el paciente no viene para el tratamiento y, por supuesto, no visita al médico. (Paciente 2)

Por lo tanto, el grado de impacto del estigma en un paciente varía según su contexto y entorno específicos. Factores como la inseguridad pública, los ingresos inestables o la lejanía geográfica pueden dar lugar a distintas experiencias de estigma, lo que diferencia a estas personas de los pacientes más afortunados.

Los participantes mencionaron con frecuencia haber experimentado efectos secundarios del tratamiento, como fatiga, sueño interrumpido, pérdida de peso, orina enrojecida, inflamación de las piernas y oscurecimiento de la piel. Estos cambios físicos a menudo llevan a los pacientes a retirarse de las interacciones sociales, lo que influye en el estigma. Sin embargo, a menudo se pasa por alto el impacto total de estos efectos relacionados con el tratamiento en el estigma. Además de los cambios visibles que provocan vergüenza, los pacientes también luchan con una fuerza física reducida, lo que limita su compromiso con el mundo exterior. Este doble impacto refuerza su aislamiento, intensificando el estigma al que se enfrentan.

## 5.1.3 Recuperación

Se considera que un paciente está libre de la enfermedad cuando la baciloscopia arroja un resultado negativo, y el seguimiento posterior se realiza anualmente durante un lapso de unos pocos años. Sin embargo, la lepra abarca dimensiones que van más allá del mero recuento bacteriano, ejerciendo efectos continuos en varias facetas de la vida que persisten después del tratamiento. Esta percepción excesivamente simplificada de la enfermedad resulta considerablemente problemática desde el punto de vista de los pacientes, dando lugar a perplejidad y exasperación. La narrativa de un participante subraya notablemente cómo el término "curado" tiene implicaciones divergentes para los trabajadores de la salud y los pacientes:

Si estoy curado, ¿por qué si me detengo, vuelvo a brotar? (...) porque no puedo decir que estoy enfermo o que estoy curado, porque solo ellos [los médicos] son los que saben, ¿no? Sin embargo, soy yo quien está sintiendo la enfermedad, ¿verdad? (Paciente 18)

Además, después del tratamiento, los pacientes a menudo requieren cuidado continuo de las heridas, calzado especializado para las deformidades del pie y deben asegurarse un sustento. Sin embargo, muchos enfrentan desafíos para regresar a sus ocupaciones anteriores debido a las discapacidades resultantes. El requisito de una evaluación clínica antes del empleo, necesaria para la cobertura del seguro, obliga a las personas a revelar información personal a extraños, una perspectiva incómoda para aquellos que evitan hablar de la lepra incluso dentro de sus familias. Además, los roles laborales físicamente exigentes a menudo los descalifican para aprobar el examen. En consecuencia, muchos participantes recurren al trabajo por cuenta propia como su única opción para mantener sus hogares.

Como enfatizó un trabajador de la salud, varios factores pueden influir en la necesidad de un subsidio por discapacidad para un paciente, incluidas las situaciones que involucran violencia doméstica. Tales circunstancias pueden afectar significativamente el proceso de evaluación y toma de decisiones con respecto a la aprobación de subsidios, como lo ejemplifica la experiencia de este paciente en particular:

Su marido la trataba mal. Ella solía decir que tenía que seguir viviendo con él porque no podía trabajar [por una discapacidad], así que le dimos un subsidio. Ella siempre está muy agradecida, y rompió con ese hombre, [la violencia doméstica] estaba complicando su situación y también estaba en constante depresión debido a su situación y a la enfermedad. (HW 4)

Este caso ilustra por qué la rígida clasificación de la enfermedad y los criterios para conceder un subsidio por discapacidad no siempre responden adecuadamente a las circunstancias individuales. Subraya la importancia de adoptar un enfoque más adaptable e inclusivo. El impacto de los factores interseccionales en la vida de un paciente puede alterar significativamente la forma en que se experimenta el estigma, lo que requiere una consideración matizada. Sin embargo, lograr esto implica brindar una atención personalizada y mantener un compromiso cercano con el paciente.

Por último, una parte crucial de la recuperación de la lepra es la integración social de los pacientes. Las asociaciones de pacientes desempeñan un papel clave mediante la educación de los miembros, la difusión de información, el fomento de las conexiones y la organización de actividades sociales. También brindan oportunidades de desarrollo de habilidades, como la artesanía, y apoyan a las pequeñas empresas, aunque han surgido desafíos, como los participantes que venden negocios para beneficio personal.

Sin embargo, en los últimos años, la asociación de pacientes ha pasado de ser una función de apoyo a convertirse en una carga financiera para algunos miembros. Las contribuciones mensuales, junto con el aumento de los gastos de transporte, sobrecargan a los pacientes que ya están lidiando con discapacidades y requieren asistencia. Además, la asociación ha comenzado a cobrar tarifas por los certificados de supervivencia, originalmente destinados a ayudar a las solicitudes de subsidios por discapacidad, pero que ahora contribuyen a la discriminación y a resultados negativos no deseados.

Las personas acuden a la asociación por obligación, por temor a que se les revoque el subsidio por discapacidad si no lo hacen (...) Les cobran 5.000 pesos por un certificado por el que no deberían cobrar. Además, algunas personas necesitan estar acompañadas por miembros de la familia debido a que están en sillas de ruedas, lo que agrega una carga económica a la familia. (Paciente 2)

## 5.2. Salud mental

En la actualidad, el sistema sanitario carece de apoyo a la salud mental y tiene dificultades para dedicar tiempo a la monitorización del bienestar de los pacientes. Por el contrario, el personal de IDS va más allá de sus funciones, ofreciendo asistencia con subsidios, diagnósticos y evaluaciones de salud mental. A pesar de las críticas por su enfoque paternalista, esta estrecha relación fomenta resultados positivos de salud mental, lo que refleja los desafíos de la incapacidad del sistema actual para brindar atención personalizada.

Es difícil que te despegues de los pacientes y les digas "no, mira, aquí no nos vemos, porque la normativa dice que no podemos". (HW 4)

Los padres entre los participantes expresaron una profunda preocupación por la transmisión de la enfermedad a sus hijos y experimentaron una pérdida de interés en interactuar con ellos, prefiriendo el aislamiento. De manera alarmante, un participante incluso intentó suicidarse, enfatizando la complejidad de abordar las necesidades de salud mental. Las barreras financieras para acceder a la medicación y al apoyo psicológico exacerban aún más estos desafíos.

Una vez intenté quitarme la vida y tomé un frasco de gotas para dormir. Lo intenté todo. Lo que quería era quedarme dormido, no volver a despertar. (Paciente 17)

La escasez de perspectivas laborales y la búsqueda de aceptación social tienen un impacto significativo en el bienestar mental de los pacientes. Después del diagnóstico, las personas a menudo pierden sus salidas habituales para la diversión, como el trabajo y los compromisos sociales, dejándolos aislados en casa, lidiando con sentimientos de insignificancia. La falta de discusiones abiertas sobre la enfermedad dentro de las familias agrava aún más este aislamiento, lo que resulta en una vida social silenciosa para los pacientes. Nos encontramos con familias con múltiples miembros afectados por la lepra, donde los mecanismos de afrontamiento se asemejaban a secretos familiares celosamente guardados. En algunos casos, la prevalencia de la enfermedad dentro de una familia incluso empañó su reputación en la comunidad. Crecer en este tipo de entornos puede convertir a las personas en personalidades reservadas e introvertidas que se contentan con estilos de vida confinados en casa.

Siempre fui tímida (...) Mi madre siempre me tuvo en casa (...) Nuestra familia [afectada por la lepra] siempre ha sido muy reservada, y no tenemos muchos amigos (...) Preferimos invitar a la familia a nuestra casa para celebrar. (Paciente 3)

## 5.3. Discriminación estructural

Nuestras entrevistas revelaron numerosos casos de discriminación y estigmatización, por parte de parejas, familiares, amigos, vecinos, empleadores o trabajadores de la salud. Sin embargo, lo que nos pareció particularmente interesante es cómo la discriminación puede rastrearse a niveles sistémicos más amplios, porque esto agrega una nueva capa a la investigación previa y subraya la necesidad de evitar un enfoque estrecho al desarrollar intervenciones para combatir el estigma.

A través del recorrido de los pacientes, se hace evidente que la discriminación está profundamente arraigada en la estructura del sistema de salud, ejerciendo su influencia en el diagnóstico, tratamiento y recuperación del paciente. Esta actitud discriminatoria imperante se manifiesta en la falta de atención y significación otorgada por el gobierno en comparación con otras enfermedades:

En el ministerio, cuando hacen reuniones de coordinadores de cada departamento [reuniones de lepra y tuberculosis], hay discriminación porque las charlas son cortas y todo el tiempo se le dedica a la tuberculosis. (HW 4)

Los cambios estructurales que se han producido en el sistema sanitario en las últimas décadas son un claro ejemplo de cómo la lepra no se considera una prioridad ni una responsabilidad, ya que la EPS ha asumido su protagonismo. La misma organización de la atención también revela sus inconvenientes en las intervenciones a largo plazo en las zonas rurales, ya que estas regiones a menudo son descuidadas y eclipsadas en comparación con las poblaciones urbanas.

Debido a la rotación personal y a las personas que hacen el año rural [año obligatorio de práctica en áreas rurales para HW], pueden pasar un año en un pueblo. Pero se acaba y cuando están aprendiendo se los quitan. (HW 4)

A lo largo de nuestras entrevistas con los trabajadores de la salud, surgió un sentimiento predominante de agotamiento en sus funciones, que identificaron como un problema importante que afectaba el diagnóstico de los pacientes. También expresaron su insatisfacción con la capacitación sobre lepra, ya que se llevó a cabo durante su horario laboral y se transfirió a plataformas en línea durante la pandemia de COVID-19. Estos trabajadores de la salud destacaron la disparidad entre el escenario ideal y los desafíos prácticos a los que se enfrentaban. En concreto, en lo que respecta al diagnóstico, describieron dificultades para comunicarse con el IDS y sentirse sin apoyo en esos momentos críticos.

El sistema consiste en que al médico se le asignan cuatro pacientes en una hora. Así, la consulta de cada paciente es de 15 minutos y a veces no es suficiente, porque si no se pone seguro contigo, no te va a decir nada. (HW 4)

El testimonio de la trabajadora de la salud pone de relieve los obstáculos para realizar diagnósticos precisos cuando los antecedentes de los pacientes no están familiarizados. Esto pone de relieve las limitaciones de las campañas de formación o concienciación por sí solas para rectificar los diagnósticos erróneos. La intrincada interacción del contexto local y la organización del sistema de salud da forma al panorama del estigma en Colombia, lo que justifica un enfoque holístico para abordar los problemas de diagnóstico erróneo. La falta crónica de fondos no solo desestabiliza los programas, sino que también conduce a un entorno de trabajo inestable, que se manifiesta en la dependencia de contratos temporales incluso para el personal de IDS. Además, la baja incidencia de la lepra complica las cosas, lo que dificulta llamar la atención sobre estas preocupaciones y puede conducir a una detección insuficiente sin esfuerzos activos de identificación de pacientes y expertos en el campo para validar las estadísticas.

La intersección de la lepra con la política es notable, con debates que se extienden a los montos de los subsidios, incluso convirtiéndose en promesas electorales. Históricamente, los pacientes participaban en actividades políticas a través de su asociación, pero su reciente disminución los deja sin representación y sus preocupaciones sin ser escuchadas. Esta pérdida de representación silencia la voz del paciente, poniendo de manifiesto la discriminación estructural a la que se enfrentan los afectados por la lepra.

# 6. Discusión

Nuestros hallazgos se alinearon con estudios previos realizados en la misma región [6] con respecto a la presencia de estigma entre los pacientes y los trabajadores de la salud. No obstante, nuestra investigación ha revelado una faceta adicional para comprender el estigma relacionado con la lepra. A lo largo de este estudio, hemos introducido una perspectiva renovada que subraya cómo los factores estructurales pueden moldear de manera intrincada la forma en que se enfrenta el estigma. Este enfoque nos ha permitido profundizar en la naturaleza multifacética del estigma relacionado con la lepra, considerando no solo las creencias individuales sino también los elementos sistémicos que contribuyen a la formación de estas experiencias.

Si bien investigaciones anteriores [6] han examinado predominantemente el estigma en los dominios individuales, relacionales y comunitarios, abarcando tanto el estigma anticipado como el internalizado, nuestro estudio examina más profundamente el estigma experimentado. Este tipo de estigma, que a menudo se observa en las relaciones interpersonales y las comunidades, justifica que se preste más atención a los intrincados mecanismos sociales que sustentan la perpetuación de la discriminación relacionada con la lepra dentro del marco estructural de Colombia.

Elegimos dividir la discusión en tres categorías: comercialización, reduccionismo bacteriológico y conocimiento del paciente. Estas categorías surgieron orgánicamente de los datos, revelando cómo se perpetúa el estigma dentro del sistema de salud colombiano.

La comercialización pone de manifiesto el paso de la medicina social a un modelo de atención sanitaria orientado al mercado, que despriorizó la lepra debido a su baja incidencia y falta de rentabilidad. Este descuido surgió cuando los participantes reflexionaron sobre cómo los programas de lepra han disminuido con el tiempo, lo que ilustra los cambios estructurales que afectan la atención al paciente.

Del mismo modo, el reduccionismo bacteriológico refleja la desconexión entre el modelo biomédico, donde la "cura" se define por la eliminación de bacterias, y las realidades vividas por los pacientes, donde siguen sufriendo efectos a largo plazo y estigma social. Nuestro análisis amplía esta desconexión y las barreras y luchas compartidas durante las entrevistas que a menudo se pasan por alto, pero que son fundamentales para comprender cómo se experimenta el estigma de la lepra.

Por último, el conocimiento del paciente tiende un puente entre las creencias locales y las explicaciones biomédicas. Con frecuencia, los participantes combinaron conocimientos científicos y culturales sobre la lepra, lo cual es crucial para comprender cómo los pacientes dan sentido a su condición en medio del estigma. Esta categoría también sirve para encapsular los razonamientos y explicaciones alternativos de la lepra compartidos por los participantes, arrojando luz sobre aspectos de la experiencia de la enfermedad que de otro modo podrían estar infravalorados, pero que son significativos.

Estas percepciones subrayan las formas en que los factores estructurales dan forma a la experiencia de la lepra más allá del estigma individual e interpersonal, lo que justifica una inclusión más amplia en el análisis del estigma relacionado con la salud.

Comercialización

Desde una perspectiva más amplia, se hizo evidente que la percepción de la enfermedad ha sufrido un proceso de comercialización, entrelazado con las transformaciones en el panorama sanitario de las últimas décadas. Hasta finales de la década de 1980, Colombia se adhirió a la tendencia latinoamericana de la medicina social, lo que resultó en una de las calificaciones de salud más admirables del continente [21]. Sin embargo, con la promulgación de la Ley 100, que reestructuró el sistema de salud para convertirlo en un mercado regulado de proveedores de seguros de salud, la trayectoria cambió hacia la privatización de la atención médica y la posterior disminución de la accesibilidad a los servicios médicos [22]. Esta trayectoria asistencial contemporánea también refleja el pasado colonial del país, influenciado por la imposición de los estándares sanitarios europeos y la profesionalización como referentes superiores [23]. Esta narrativa de comercialización podría ser indicativa de un continuo de dinámicas de poder coloniales, donde los procesos de globalización y la preferencia por modelos de salud extranjeros han perpetuado aún más la tendencia.

La lepra, caracterizada por su baja tasa de incidencia y la continua necesidad de recursos humanos y financieros para la identificación de casos y el seguimiento de los pacientes, ha sido víctima de un mayor abandono en el contexto del sistema de salud. Este desconocimiento puede atribuirse a la ausencia de incentivos comerciales tanto para el SPA como para el IPS. Impulsadas por un análisis costo-beneficio, estas entidades tienden a limitar sus servicios, acortar los tiempos de consulta y priorizar las necesidades de la población sana sobre las de los pacientes con lepra [24].

Nuestros resultados se alinean con otros estudios en Colombia [25] que subrayan la importancia de la transmisión familiar de la lepra como un modo primario de propagación de la enfermedad. Este hallazgo refuerza la idea de que la estrecha vigilancia de los contactos en el hogar es una estrategia valiosa tanto para el diagnóstico precoz de la lepra como para la vigilancia de su dinámica de transmisión. Sin embargo, la trayectoria prevaleciente del sistema de salud del país no está alineada con este tipo de estrategias proactivas. La dirección actual del sistema de salud no prioriza estos enfoques, lo que podría tener un impacto potencial en los esfuerzos de control de la lepra. Los hallazgos enfatizan la importancia de cerrar esta brecha entre los conocimientos de la investigación y las políticas de atención médica para abordar de manera efectiva los desafíos que plantea la transmisión de la lepra y sus complejidades asociadas.

La transformación de la asistencia sanitaria en una esfera comercializada también ha alterado el enfoque de la gestión de la lepra, pasando de un modelo caritativo o paternalista a una forma de atención más medicalizada e impersonal. En consecuencia, esta transformación ha llevado a una reducción en el alcance de la participación de los trabajadores de la salud, con menos énfasis en el apoyo continuo al paciente a lo largo de su viaje con la enfermedad. Como resultado, se han pasado por alto y olvidado varias facetas de la lepra más allá de los aspectos clínicos, socavando así iniciativas como la asociación de pacientes mencionada en los resultados. Se hace evidente que este enfoque comercializado ha contribuido a que la asociación de pacientes con lepra pierda su capacidad para representar eficazmente a los pacientes y fomentar su inclusión, perdiendo las conexiones que una vez cerraron la brecha entre los pacientes antiguos y los nuevos.

El examen de las experiencias pasadas de los participantes con la asociación proporciona una perspectiva interesante. Si bien los resultados del estigma se consideran comúnmente negativos, vale la pena señalar que también pueden surgir resultados positivos. Se ha reconocido que el estigma fomenta la resiliencia entre los grupos marginados [26], y ha desempeñado un papel en la catalización del establecimiento de grupos y campañas de defensa de los pacientes. Estos esfuerzos han contribuido a cambios significativos en las políticas destinadas a mejorar el acceso a la atención médica para enfermedades estigmatizadas, como el VIH [27]. En el caso de la lepra, este fue el caso en el pasado, desafiando la noción demasiado simplificada de que las poblaciones vulnerables están definidas y conectadas únicamente por su vulnerabilidad percibida. Esto pone de manifiesto la naturaleza fluida y compleja de cómo se conceptualiza a los pacientes [28].

Reduccionismo bacteriológico

El contraste entre la perspectiva biomédica de la lepra como un simple recuento bacteriológico y la comprensión holística de los pacientes ejemplifica las repercusiones del proceso de comercialización. La erradicación del bacilo de Hansen ha llevado a la percepción de que un paciente ya no se considera enfermo, pasando por alto los efectos duraderos del tratamiento, los impactos persistentes de la lepra y los desafíos continuos para acceder al tratamiento y mantener los medios de vida. En consecuencia, la noción binaria convencional de enfermedad y salud se vuelve problemática cuando se pone en práctica.

La negativa del paciente a abrazar plenamente el concepto de cura bacteriológica está intrínsecamente ligada a su resistencia a ser categorizado como "socialmente curado". Esta categoría fue introducida por el Estado colombiano en la década de 1920 para etiquetar a aquellos individuos que ya no eran considerados una amenaza para la sociedad debido a su mejor estado de salud. Los que caían en esta categoría fueron dados de alta de los leprarios y se puso fin a sus subsidios, ya que se esperaba que se reintegraran a la sociedad. Sin embargo, este enfoque reduccionista de la enfermedad fue impulsado principalmente por consideraciones económicas para reducir el costo de mantener los leprosos cuando el confinamiento era obligatorio [22; 29]. Los mismos conflictos y desafíos del contexto histórico de la lepra continúan reverberando en la actualidad.

La perspectiva reduccionista enraizada en el análisis bacteriológico no se limita a la fase de recuperación del paciente, sino que también se extiende al proceso diagnóstico. En la búsqueda de hacer que las técnicas biomédicas como las biopsias y las baciloscopias funcionen, otros métodos valiosos como los exámenes físicos y la evaluación integral de la historia clínica pierden su importancia. Cuando estos métodos biomédicos fallan debido a errores humanos o tiempos de espera prolongados (el retraso promedio en el diagnóstico en Colombia es de 2,7 años [18]), los pacientes se quedan sin alternativa para el diagnóstico, incluso si sus síntomas son evidentes para un médico experimentado. De hecho, el hecho de que no se reconozcan y consideren otras dimensiones de la enfermedad más allá del mero recuento bacteriológico, junto con el desprecio por la importancia de los factores interseccionales en la comprensión de la lepra y su estigma asociado, contribuye directamente a un sistema de discriminación estructural. Al no incorporar una comprensión más amplia que abarque los elementos socioeconómicos, culturales y contextuales de la lepra, el sistema de salud perpetúa inadvertidamente las disparidades y las barreras, lo que en última instancia profundiza el impacto del estigma y obstaculiza las intervenciones efectivas.

Pasar por alto factores como la ubicación geográfica del paciente o la situación de seguridad local puede llevar a una comprensión superficial de cómo se experimenta el estigma relacionado con la lepra. Este enfoque puede pasar por alto las intrincadas conexiones entre el estigma de la lepra y las formas más amplias de marginación, como las basadas en la raza, la clase y el género. En consonancia con investigaciones previas [27], nuestros hallazgos enfatizan que el estigma de la lepra interactúa con estas otras capas de desventaja, lo que resulta en diversos grados de vulnerabilidad al estigma relacionado con la salud. Nuestro estudio se hace eco de los hallazgos [30] que destacan el impacto del estatus socioeconómico en la experiencia del estigma relacionado con la salud, enfatizando particularmente la pobreza como un desafío interseccional significativo. Si no se tienen en cuenta estos factores intrincados, se puede exacerbar la carga de la lepra, lo que puede provocar interrupciones en el tratamiento y resultados negativos. Para abordar de manera integral las complejidades del impacto de la lepra, es crucial reconocer y abordar los factores entrelazados que contribuyen al estigma, guiando el desarrollo de intervenciones que aborden los obstáculos multifacéticos que enfrentan los pacientes.

Teniendo en cuenta las luchas históricas por la autoridad profesional entre los médicos en Colombia [23], es interesante observar cómo los médicos contemporáneos priorizan mantener su estatus de confianza y confiabilidad. Este énfasis en la credibilidad puede ser visto como una extensión del legado actual de la medicalización, que, como destaca Platarrueda Vanegas [29], sigue siendo incompleto debido a los diálogos rotos y a la exclusión del conocimiento experiencial. La narrativa médica se cruza con otras dimensiones de la enfermedad en la comprensión de la lepra por parte del paciente. El concepto de reduccionismo bacteriológico [29], en el que se considera que un paciente está curado únicamente por la finalización del tratamiento, contrasta fuertemente con las incertidumbres y frustraciones del paciente, ya que no se percibe a sí mismo como completamente curado. Esto se agrava aún más cuando el paciente sufre reacciones de lepra. Desafortunadamente, ahora que la lepra multibacilar se trata en gran medida con un año de terapia multifarmacológica, los estados de reacción pueden continuar después de la finalización de la terapia [31].  Por lo tanto, los aspectos complejos de la enfermedad crean espacio para interpretaciones variadas y puntos de vista alternativos que deben integrarse junto con el enfoque biomédico.

Además, el frecuente diagnóstico erróneo de la lepra por parte de médicos que carecen de tiempo suficiente para familiarizarse con los pacientes o que con frecuencia son rotados a diferentes clínicas erosiona la confianza en su función. Por el contrario, los no profesionales experimentados que poseen un conocimiento más profundo de la lepra a menudo intervienen para cerrar esta brecha en el sistema, debido a una capacitación insuficiente o al agotamiento de los médicos. Estos pacientes experimentados se convierten en las principales fuentes de información y diagnóstico para los nuevos pacientes. En consecuencia, la autoridad de los médicos se ve socavada en tales situaciones, lo que genera conflictos y compromete la comunicación efectiva con los pacientes. Este escenario subraya la importancia de abordar las deficiencias del sistema médico para mejorar las relaciones médico-paciente y los resultados generales de la atención médica.

Conocimiento del paciente

De acuerdo con la literatura existente que sugiere que los pacientes adoptan varias explicaciones para la causalidad de la lepra para mitigar el miedo social [32], nuestro estudio revela que los participantes también incorporan teorías de herencia, estilo de vida, nutrición, medio ambiente y condiciones sociales para contextualizar los orígenes de la lepra. Curiosamente, algunos participantes atribuyeron la aparición de la enfermedad a factores como deficiencias inmunológicas, condiciones insalubres, hábitos dietéticos, climas cálidos u ocupaciones poco saludables. Este entrelazamiento de narrativas biomédicas y experienciales se hace evidente cuando se examinan los orígenes de la naturaleza endémica de la lepra. Los trabajadores de la salud también reconocen los cambios inmunológicos y las condiciones desfavorables como factores que contribuyen al desarrollo de la lepra, lo que valida las creencias locales. Esta convergencia de razonamientos locales y conocimientos biomédicos demuestra una compleja interacción de explicaciones que buscan racionalizar la aparición de la lepra, ofreciendo un puente entre las interpretaciones culturales y las comprensiones médicas.

La religión, históricamente entrelazada con la percepción de la lepra, sigue teniendo un papel en la comprensión contemporánea de la enfermedad. Tradicionalmente asociada con el pecado y el castigo, la lepra tenía connotaciones estigmatizantes [33]. Sin embargo, nuestro estudio no reveló tales asociaciones negativas en las entrevistas de los participantes. En cambio, la religión sirve como un marco para dar sentido a la lepra, dando a Dios la autoría de los descubrimientos científicos y viendo la fe como una fuente de esperanza y consuelo. Esta interpretación de la lepra impulsada por el paciente enfatiza la complejidad de sus percepciones culturales y médicas, ofreciendo información sobre las formas matizadas en que las personas contextualizan y explican sus experiencias con la enfermedad.

La incorporación de la construcción médica del estigma en nuestro análisis nos llevó a encontrarnos con ciertas situaciones que no encajaban perfectamente en las categorizaciones establecidas. Por ejemplo, la renuencia de los participantes a revelar su historial médico a los médicos podría percibirse como una forma de estigma anticipado por las definiciones médicas [6]. Sin embargo, nuestros hallazgos revelaron que algunos participantes optaron por no revelar sus antecedentes de lepra a los médicos para asegurar un diagnóstico no relacionado con la lepra, aprovechándose de la falta de conocimiento de los médicos. Este comportamiento se ha observado en la obesidad, donde los pacientes ocultan información para evitar prejuicios y el daño iatrogénico asociado a un diagnóstico inexacto [34]. En este contexto, la lepra se entrelaza con otras enfermedades, impactando en el proceso de diagnóstico y en la relación médico-paciente.

Desde el punto de vista del paciente, donde el tiempo con el médico a menudo es limitado, evitar mencionar la lepra puede simplificar el diagnóstico. Estas experiencias ponen de manifiesto una forma distintiva de construcción del estigma en Colombia, donde las consideraciones y estrategias empleadas por los pacientes para navegar por el sistema de salud difieren. Reconocer y comprender esta conceptualización única del estigma es esencial para diseñar intervenciones efectivas que se adapten a los desafíos específicos que enfrentan los pacientes en Colombia.

Al examinar las experiencias de los pacientes, surgió un énfasis significativo en la salud mental y el subsidio por discapacidad. Estos factores fueron temas recurrentes e interconectados que aparecieron en diferentes categorías durante nuestro análisis. A pesar del impacto bien documentado y medido de la lepra en el bienestar mental, es notable que las consideraciones de salud mental a menudo no se incluyen en la evaluación más amplia de la carga de la enfermedad [35]. Nuestros hallazgos se alinearon con estudios previos [6] que destacaron una carga psicosocial sustancial experimentada por los pacientes con lepra. En la región de Norte de Santander, este aspecto parece pasarse por alto, ya que muchos participantes describieron una realidad en la que los pacientes luchan contra la depresión y la ansiedad sin el apoyo suficiente. El acceso a recursos adecuados de salud mental se ve obstaculizado por barreras como el costo de los medicamentos y la ayuda profesional. Son muchos los factores que se entrelazan con la salud mental de los pacientes: el intrincado y largo viaje de la lepra, los efectos secundarios del tratamiento, la inestabilidad económica y social, la realización personal y la experiencia del aislamiento social. Reconocer y abordar estos desafíos de salud mental es vital para brindar atención y apoyo integrales a los pacientes con lepra en Colombia.

El subsidio por discapacidad es un factor interseccional adicional que impacta positivamente en el bienestar mental de un paciente al proporcionar una sensación de seguridad y estabilidad. Sin embargo, el proceso de obtención, gestión y renovación del subsidio plantea desafíos y conflictos. En primer lugar, la elegibilidad para el subsidio está determinada por categorías fijas predefinidas, como el nivel de discapacidad e ingresos, que simplifican demasiado las realidades del paciente. Por lo tanto, se pasan por alto casos que deben ser considerados para recibir apoyo, como el caso de una paciente que sufre violencia doméstica y depende de su agresor para sobrevivir. En segundo lugar, el subsidio del paciente tiene implicaciones significativas para la situación financiera de su familia que, cuando no se controla, coloca al paciente en una posición vulnerable. La implementación de programas de seguimiento, educación financiera y asistencia en la gestión del subsidio es crucial para prevenir la explotación y garantizar los beneficios previstos. En tercer lugar, el requisito del gobierno de demostrar la supervivencia del paciente para mantener el subsidio pone de manifiesto cómo el entorno burocrático puede complicar las cosas. En respuesta, la asociación de pacientes ha asumido el papel de ayudar a los pacientes en este proceso. Desafortunadamente, este acuerdo obliga a los pacientes a contribuir financieramente a la asociación, lo que puede crear una carga financiera adicional para ellos. Abordar estas complejidades y desafíos asociados con el subsidio requiere un enfoque holístico que considere las diversas circunstancias de los pacientes y busque brindar un apoyo significativo sin sobrecargarlos aún más.

La ausencia de incentivos para involucrar a pacientes expertos como trabajadores sociales contrasta completamente con los enfoques históricos del tratamiento de la lepra, lo que deja un vacío significativo en la comprensión y el tratamiento de la enfermedad. Estas personas poseen conocimientos invaluables derivados de su experiencia de primera mano de vivir con lepra. Su perspectiva podría aprovecharse para actividades como la detección de casos, los procedimientos de seguimiento y las campañas de información. Poseen una comprensión íntima de los desafíos emocionales y prácticos que enfrentan los pacientes, percepciones que a menudo son mejor comprendidas por aquellos que las han vivido. Sus perspectivas únicas se extienden a aspectos como el impacto psicológico de crecer con lepra, cómo moldea la personalidad y las barreras cotidianas que enfrentan los pacientes.

La integración de narrativas alternativas con el marco biomédico es insuficiente, lo que pone de manifiesto la necesidad de un diálogo más fuerte que fusione estos paradigmas. Cerrar esta brecha entre los enfoques biomédicos y las experiencias de los pacientes es crucial para una comprensión holística de la lepra más allá de los contextos históricos. Esta integración puede conducir a estrategias de manejo de enfermedades más integrales, diseñadas con sensibilidad a los desafíos multifacéticos de los pacientes.

Los esfuerzos eficaces para combatir el estigma relacionado con la lepra requieren un enfoque integral que tenga en cuenta el contexto social más amplio y la intrincada interacción de diversos factores. Es esencial incorporar las experiencias y las voces de las personas afectadas por la lepra, así como abordar las desigualdades estructurales que contribuyen al estigma. Al adoptar una visión holística y trabajar para desmantelar las barreras estructurales que sostienen el estigma, es posible crear estrategias más inclusivas y efectivas que realmente apoyen a los pacientes de lepra y su bienestar.

## Limitaciones

Este estudio tiene especial importancia dada la naturaleza endémica de la lepra en el área investigada. Sin embargo, aún existe la necesidad de ampliar el alcance geográfico de este estudio para incorporar plenamente la complejidad y diversidad de Colombia. Vale la pena señalar que el alcance del estudio estuvo limitado por las fronteras nacionales con Venezuela. Sin embargo, es importante reconocer que las enfermedades no respetan fronteras, lo que amerita una consideración más profunda de las áreas circundantes de Norte de Santander. Esas regiones pueden actuar como encrucijadas importantes para la transmisión de enfermedades debido a los movimientos transfronterizos. Además, el examen de estas regiones pone de relieve la importancia de los esfuerzos de colaboración transfronterizos para gestionar eficazmente el control de las enfermedades.

# 7. Conclusión

Esta investigación amplía el enfoque del estigma relacionado con la lepra para revelar elementos estructurales del estigma en el sistema de salud colombiano. Destaca cómo el abandono de la lepra es evidente en la organización, la asignación de recursos, la atención prestada y los desafíos que enfrentan los trabajadores de la salud. En conclusión, la comercialización de la salud, el reduccionismo bacteriológico, la omisión de las narrativas de los pacientes y el paso por alto de diversos desafíos y factores interseccionales a lo largo del viaje de la lepra son indicadores claros de estigma y discriminación arraigados a niveles estructurales. Este sesgo sistémico permea todos los niveles de la sociedad colombiana. No reconocer y abordar esta realidad a la hora de elaborar intervenciones, tratamientos, políticas o investigaciones perpetúa y refuerza el estigma existente en torno a la lepra.

Las recomendaciones incluyen la participación de los pacientes en la planificación del programa, la integración de los sistemas de historia clínica, la detección proactiva de casos, el tratamiento específico del contexto y la participación del paciente en iniciativas públicas. Estas medidas tienen como objetivo empoderar a los pacientes, mejorar el bienestar mental y reducir el estigma dentro de la sociedad y las instituciones colombianas.

# Reconocimientos

Estoy profundamente agradecido al equipo de DAHW en Colombia por su excepcional trabajo en la lucha contra la lepra y por su cálida bienvenida y asistencia a lo largo de esta investigación. Por último, estoy profundamente agradecido a los pacientes que amablemente compartieron sus conocimientos y experiencias, inspirándome con su resiliencia y generosidad. Sin su apoyo y contribuciones colectivas, esta investigación no habría sido posible.

**Consentimiento para la publicación:** Todos los autores leyeron y aprobaron el manuscrito final.

**Intereses contrapuestos:** Los autores declaran que no tienen intereses contrapuestos.

Aportaciones de los autores: AF y CMG diseñaron el estudio. CMG realizó las entrevistas y analizó los datos. CG guió a CMG a través del proceso de entrevista y análisis. CMG proporcionó el primer borrador del manuscrito. CG y CMG contribuyeron al manuscrito final. AF supervisó el estudio. Todos los autores leyeron y aprobaron el manuscrito final.

# Referencias

1. Organización Mundial de la Salud. Lepra [Internet]. 2023 [citado 27 ene 2023]. Disponible en: [https://www.who.int/news-room/fact-sheets/detail/leprosy](https://www.who.int/news-room/fact-sheets/detail/leprosy)
2. White C, Franco-Paredes C. La lepra en el siglo XXI. Clin Microbiol Rev. 2015; 28(1): 80-94. doi: 10.1128/CMR.00079-13
3. Dharmawan Y, Fuady A, Korfage IJ, Richardus JH. Detección tardía de casos de lepra: una revisión sistemática de los factores relacionados con la atención médica. PLoS Negl Trop Dis. 2022; 16(9):E0010756. doi: 10.1371/journal.pntd.0010756
4. Cardona-Castro, Nuevo México. Lepra en Colombia: etapa post eliminación. Lepr Rev. 2013; 84:238–247.
5. Gómez LJ, Van Wijk R, Van Selm L, Rivera A, Barbosa MC, Parisi S, et al. Estigma, restricción de la participación y sufrimiento mental en pacientes afectados por lepra, leishmaniasis cutánea y enfermedad de Chagas: un estudio piloto en dos regiones coendémicas del oriente colombiano. Trans R Soc Trop Med Hyg. 2020; 114(7):476–482. doi: 10.1093/trstmh/trz132
6. van Wijk R, van Selm L, Barbosa MC, van Brakel WH, Waltz M, Puchner KP. Carga psicosocial de las enfermedades tropicales desatendidas en el oriente colombiano: un estudio cualitativo exploratorio en personas afectadas por lepra, leishmaniasis cutánea y enfermedad de Chagas. Salud Mental Global. 2021; (Mateo 8:E21:1–7). doi: 10.1017/gmh.2021.18
7. Dako-Gyeke M, Asampong E, Oduro R. Estigmatización y discriminación: Experiencias de personas afectadas por la lepra en el sur de Ghana. Lepr Rev. 2017; 88(1):58–74.
8. Rai SS, Peters RMH, Syurina EV, Irwanto I, Naniche D, Zweekhorst MBM. Interseccionalidad y estigma relacionado con la salud: perspectivas de las experiencias de personas que viven con condiciones de salud estigmatizadas en Indonesia. Int J Equidad Salud. 2020; 19(1):206. DOI: 10.1186/s12939-020-01318-W
9. van Brakel WH, Cataldo J, Grover S, et al. Fuera de los silos: identificación de las características transversales del estigma relacionado con la salud para avanzar en la medición y la intervención. BMC Med. 2019;17:13. DOI: 10.1186/s12916-018-1245-x
10. Gómez LJ, Rivera A, Vidal Y, Bilbao J, Kasang C, Parisi S, et al. Factores asociados al retraso en el diagnóstico de la lepra en el noreste colombiano: un análisis cuantitativo. Trop med int salud. 2018; 23(2):193–198. doi: 10.1111/tmi.13023
11. Staples, J. Nuancing: "El estigma de la lepra" a través de una biografía etnográfica en el sur de la India. Lepr Rev. 2011; 82(2):109-123. doi: 10.47276/lr.82.2.109.
12. Weiss MG, Ramakrishna J, Somma D. Estigma relacionado con la salud: replanteamiento de conceptos e intervenciones. Psychol Health Med. 2006; 11(3):277–287. doi: 10.1080/13548500600595053
13. Bijker NOSOTROS. Comprender la cultura tecnológica a través de una visión constructivista de la ciencia, la tecnología y la sociedad. En: Cutcliffe SH, Mitcham C, editores. Visiones de SAS; contrapuntos en los Estudios de la Ciencia, la Tecnología y la Sociedad. Nueva York: Editorial de la Universidad Estatal de Nueva York; 2001.
14. Obregón D. La construcción social de la lepra en Colombia, 1884-1939. Sci Technol Soc. 1996; 1(1):1–23. doi: 10.1177/097172189600100102
15. Bronfenbrenner U. Modelos ecológicos del desarrollo humano. En: Enciclopedia internacional de la educación. Oxford, Inglaterra: Elsevier; 1994.
16. Thorogood N, Green J. Métodos cualitativos para la investigación en salud. Salvia; 2018.
17. Saldaña, J. Manual de codificación para investigadores cualitativos (2ª ed.). Londres: Sage; 2013.
18. Tolley EE, Ulin PR, Mack N, Robinson ET, Succop SM. Métodos cualitativos en salud pública: una guía de campo para la investigación aplicada (Second, Ser. Jossey-bass public health). Wiley; 2016.
19. Gómez L, Rivera A, Vidal Y, Bilbao J, Kasang C, Parisi S, et al. Factores asociados al retraso en el diagnóstico de la lepra en el noreste colombiano: un análisis cuantitativo. Trop med int salud. 2018; 23(2):193–198. doi: 10.1111/tmi.13023
20. Deps P, Antunes JMAP, Collin SM. Riesgo zoonótico de la enfermedad de Hansen por el contacto de la comunidad con armadillos salvajes: una revisión sistemática y metaanálisis. Zoonosis Salud Pública. marzo de 2021; 68(2):153-64. doi: 10.1111/zph.12783. Epub 23 de noviembre de 2020. PMID: 33226194.
21. Abadía-Barrero CE. Políticas y sujetos del sida en Brasil y Colombia. Rev Colomb Antropol. 2004;40:123-154.
22. Ahumada C. La penuria de la salud pública. Rev Gerenc Polit Salud. 2002;47-56.
23. Obregón D. Construyendo la medicina nacional: lepra y poder en Colombia, 1870-1910. Soc Hist Med. 2002; 15(1):89–108. doi: 10.1093/shm/15.1.89
24. Laurell AC, López Arellano O. Productos básicos de mercado y alivio a los pobres: La propuesta del Banco Mundial para la salud. Int J Health Serv. 1996; 26(1). doi: 10.2190/PBX9-N89E-4QFE-046V
25. Romero-Montoya M, Beltrán-Alzate JC, Cardona-Castro N. Evaluación y monitoreo de la transmisión de Mycobacterium leprae en contactos domiciliarios de pacientes con enfermedad de Hansen en Colombia. PLoS Negl Trop Dis. 2017; 11(1):E0005325. doi: 10.1371/journal.pntd.0005325
26. Trapence G, Collins C, Avrett S, Carr R, Sanchez H, Ayala G, et al. De la supervivencia personal a la salud pública: liderazgo comunitario de hombres que tienen sexo con hombres en la respuesta al VIH. The Lancet. 2012. doi: 10.1016/S0140-6736(12
27. Stangl, A. L., Earnshaw, V. A., Logie, C. H., van Brakel, W., C Simbayi, L., Barré, I., & Dovidio, J. F. (2019). El Marco del Estigma y la Discriminación en la Salud: un marco global y transversal para informar la investigación, el desarrollo de intervenciones y las políticas sobre los estigmas relacionados con la salud. BMC medicina, 17(1): 31. doi: 10.1186/s12916-019-1271-3
28. Kippax, S., Stephenson, N., Parker, R. G., & Aggleton, P. (2013). Entre la agencia individual y la estructura en la prevención del VIH: comprensión del punto medio de la práctica social. Revista Americana de Salud Pública, 103(8), 1367-1375. doi: 10.2105/AJPH.2013.301301
29. Platarrueda Vanegas, C. (2007). La voz del proscrito o la exclusión desde adentro: lepra y representaciones sociales de los lazaretos en Colombia. Una aproximación antropológica [Tesis doctoral, Universidad Nacional de Colombia]. Disponible en: https://www.humanas.unal.edu.co/2017/investigacion/application/files/2115/5665/418 8/Pre- La_voz_del_proscrito._Experiencia_de_la_lepra_y_devenir_de_los_lazaretos_en_Col ombia.pdf
30. Rai, S. S., Peters, R. M. H., Syurina, E. V., Irwanto, I., Naniche, D., & Zweekhorst, M. B. M. (2020). Interseccionalidad y estigma relacionado con la salud: perspectivas de las experiencias de personas que viven con condiciones de salud estigmatizadas en Indonesia. Revista internacional de equidad en salud, 19(1): 206. DOI: 10.1186/s12939-020-01318-W
31. Balagon MV, Gelber RH, Abalos RM, Cellona RV. Reacciones después de completar la terapia multifármaco (MDT) de 1 y 2 años. Am J Trop Med Hyg. septiembre de 2010; 83(3):637-44. doi: 10.4269/ajtmh.2010.09-0586. PMID: 20810832; PMCID: PMC2929063.
32. Blanco, C. (2002). Consideraciones socioculturales en el tratamiento de la lepra en Río de Janeiro, Brasil. Revista de lepra, 73(4), 356–365.
33. Botero-Jaramillo, N., Rivas, D. P., & Rueda L. S. (2015). La lepra en Colombia: estigma, identidad y resistencia en los siglos XX y XXI. Revista Salud Bosque. 5(1), 67-80. doi: 10.18270/rsb.v5i1.185
34. Brewis A, Wutich A. Perezoso, loco y repugnante: el estigma y la perdición de la salud global. Baltimore: Johns Hopkins University Press; 2019
35. Somar, P., Waltz, M. M., & van Brakel, W. H. (2020). El impacto de la lepra en el bienestar mental de las personas afectadas por la lepra y sus familiares: una revisión sistemática. Salud mental global, 7, e15. doi: 10.1017/gmh.2020.3
